# Evidence for Competing Proton-Coupled Reaction Pathways of Molecular Triads in a Low-Polarity Solvent

**DOI:** 10.1021/acs.jpca.4c05734

**Published:** 2025-02-06

**Authors:** Laura F. Cotter, Giovanny A. Parada, Rohit Bhide, Belinda Pettersson Rimgard, James M. Mayer, Leif Hammarström

**Affiliations:** † Department of Chemistry, Yale University, New Haven, Connecticut 06520, United States; ‡ Department of Chemistry, The College of New Jersey, Ewing, New Jersey 08628, United States; § Department of Chemistry − Ångström Laboratory, Uppsala University, Box 523, SE75120 Uppsala, Sweden

## Abstract

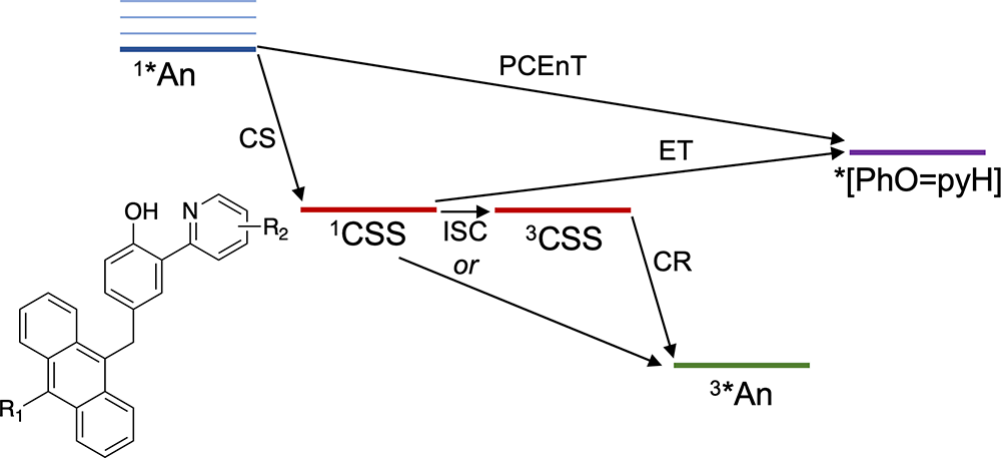

The temperature dependence of concerted proton-electron transfer (CPET) reactions of two anthracene-phenol-pyridine (An-PhOH-py) triads is investigated in toluene. Light excitation forms an anthracene local excited state (^1^*^^An), which undergoes CPET to form a charge separated state (CSS, An^•–^-PhO^•^-pyH^+^), which in turn undergoes CPET charge recombination (CR). In toluene, compared with polar solvents, the CSS is energetically destabilized. First, this makes another reaction competitive with CPET, which we propose is proton-coupled energy transfer (PCEnT) from ^1^*^^An to form the short-lived excited state keto tautomer of the phenol-pyridine subunit (*[PhO=pyH]). Second, it puts CR deep into the Marcus inverted region, and CSS lifetimes therefore reach several nanoseconds at room temperature. The slow kinetics makes CR to the anthracene triplet state (^3^*^^An) competitive, as well as another reaction that is strongly activated and dominates CSS deactivation at *T* ≥ 240 K for one of the triads. The latter is proposed to be CR via initial formation of the same [*PhO=PyH] state as above by an unusual electron transfer (ET) from An^•–^ to pyH^+^, instead of CR with the juxtaposed PhO^•^. The two different pathways to form *[PhO=pyH] lead to CSS yields and lifetimes that vary significantly with temperature, and in markedly different ways between the triads. This is rationalized by the differences in the energies of the states involved. The results broaden the scope and understanding of the still rare phenomena of inverted CPET and PCEnT and may aid toward their use in solar fuels and photoredox catalysis.

## Introduction

**Figure 1:**
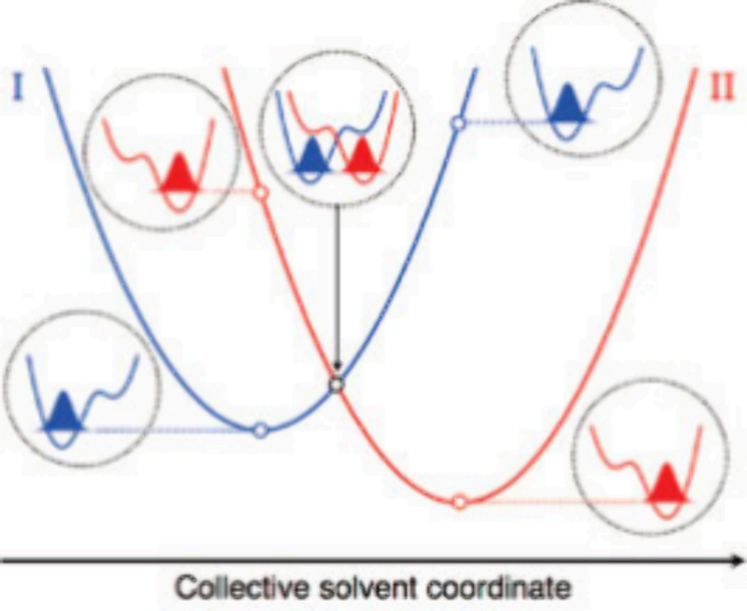
Free energy curves for the ground reactant (blue) and product (red) states along a collective solvent coordinate. Proton potential energy surfaces and vibrational wave functions at selected points along the reactant and product surfaces. Reproduced with permission from ref (10). Copyright 2008 American Chemical Society.

Proton-coupled electron transfer (PCET) is a fundamental mechanism in chemical and biological processes such as energy conversion, redox catalysis, photoreceptor triggering, and DNA synthesis and repair.^1−8^ PCET reactions comprise two elementary types of charge transfer: proton transfer (PT) and electron transfer (ET). During a PCET reaction, the proton and electron can transfer either sequentially or in a single, concerted step. The latter scenario is known as concerted proton-electron transfer (CPET; EPT and CEPT are also common abbreviations with the same meaning) and includes hydrogen atom transfer as well as reactions in which electrons and protons are transferred to or from different sites. A CPET mechanism is the more energetically favorable pathway to PCET products, as it bypasses the high-energy, often charged, intermediates that would be formed via a sequential mechanism. Current theories describe CPET as a Marcus-type charge transfer reaction where solvent and other heavy nuclei need to reorganize to reach resonance of reactant and product states, at which point both electron and proton tunnel from donor to acceptor (Figure 1).^7,8^ The resulting nonadiabatic rate expression in eq 1 includes the sum of contributions from all proton vibrational states in reactant (μ) and product (ν). The theory has been quite successful in analyzing experimental data. Still, the free-energy dependence predicted by eq 1, including the inverted region where the rate constant decreases as the driving force increases, was experimentally verified only recently.^9^
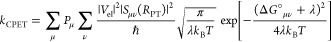
1

**Figure 2:**
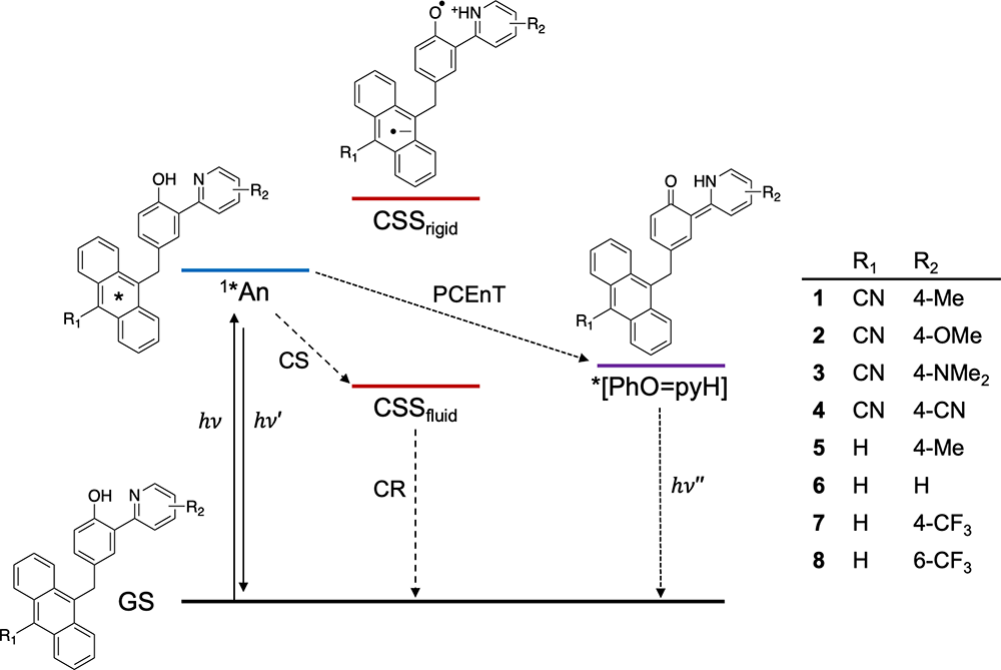
Structures of anthracene-phenol-pyridine (An-PhOH-py) triads, **1**–**8**, and Jablonski diagram of relevant states and transitions observed for **1**–**3** in polar solvents such as butyronitrile in fluid solution at 298 K (dashed arrows) and in a rigid glass at 77 K (dotted arrows).^9,12^ Under all conditions studied, excitation with 400 nm light excites the anthracene moiety to form a local excited state (^1^*^^An). In fluid solution at 298 K, ^1^*^^An undergoes a CPET charge separation (CS) reaction that forms the charge separated state (CSS). The CSS reforms the ground state (GS) by the CPET charge recombination (CR) reaction, the rate of which has an inverted dependence on driving force. Alternatively, in a rigid medium (such as a 77 K glass), the CSS is destabilized and energetically inaccessible, and PCEnT from ^1^*^^An can populate the excited keto tautomer form of the phenol-pyridine unit (*[PhO=pyH]) that is observed by its fluorescence.

Our groups have reported on the photochemical PCET reactions of a series of anthracene-phenol-pyridine (An-PhOH-py) triads **1**–**8** (Figure 2).^9,11−13^ Despite their structural similarities, **1**–**8** display a range of photochemical processes. Variable substituents on the anthracene and pyridine subunits alter the driving forces for photoinduced PCET processes. For all triads, selective excitation of the anthracene forms a local excited state (^1^*^^An). From ^1^*^^An, **1**–**3** vs **4**–**8** diverge in their pathways to return back to the ground state. **1**–**3** undergo ET from the phenol to ^1^*^^An concerted with PT from the phenol to the pyridine in solvents of medium to high polarity and over a wide range of temperatures.^9,12^ This CPET charge separation (CS) results in the formation of the charge-separated state (CSS,An^•–^-PhO^•^-pyH^+^). The CSS then undergoes CPET charge recombination (CR) reforming the ground state and completing the photochemical cycle. The rates of CPET CR for **1**–**3** show the first example of an inverted region dependence on driving force probed by substituent effects and solvent polarity.^9^ Under similar conditions, however, **4**–**8** do not show the spectroscopic signatures of the CSS, and therefore, other pathways are suspected to be involved in their deactivation.

In rigid media, where the CSS is destabilized, thus blocking PCET on thermodynamic grounds, we later found that both groups of triads undergo a new type of reaction that we denoted proton-coupled energy transfer (PCEnT; Figure 2).^13^ In PCEnT, energy transfer and PT occur in a single concerted reaction step to form the excited state proton-transfer keto tautomer of the phenol-pyridine unit (*[PhO=pyH]) directly from ^1^*^^An; the mechanism of PCEnT is discussed below. The resulting *[PhO=pyH] state is observed by its fluorescence in rigid media, and it forms without population of the higher-lying excited enol form (*[PhOH-py]). The involvement of *[PhO=pyH] in the excited state dynamics of the triads was initially suggested by computational studies^14,15^ and can explain the differences in behavior between **1**–**3** vs **4**–**8** in fluid solution. To investigate the factors that influence the preference for CPET CS vs PCEnT, we decided to study **1** and **2** in toluene as a low-polarity solvent in fluid media between 185 and 298 K where the CSS is destabilized but still below ^1^*^^An.

Here we show competing photoinduced reactions, by temperature dependence studies, that we assign to proton-coupled energy vs electron transfer in toluene for **1** and **2**. As expected, the low polarity solvent drives the CPET CR from the CSS to the ground state deeper into the Marcus inverted region. The effect extends the CSS lifetime, and for **2**, the CSS persists for up to several nanoseconds at room temperature—a slow time scale suitable to coupling with other chemical reactions, especially processes that are relevant to solar fuels and photoredox catalysis. In toluene, the CSS is destabilized to an extent that the proposed PCEnT and CPET CS from ^1^*^^An in **1** occur on similar time scales, depending on the temperature. Distinctly different CSS yields and lifetimes are observed for **1** versus **2** when varying the temperature, which contrasts the similar temperature dependencies reported in nitrile solvents.^12^ The results suggest that ^1^*^^An deactivates by PCEnT to form *[PhO=pyH] in fluid media, just as in low-temperature glass,^13^ in competition with PCET to form the CSS. This is inferred from relative yields, lifetimes, and temperature and substituent dependences, as *[PhO=pyH] deactivates to the ground state on an ultrafast time scale in fluid solution and cannot be directly observed under the present experimental conditions. Notably, we find evidence suggesting that *[PhO=pyH] is also populated from the CSS by ET from An^•–^ to (formally) the pyH^+^ unit in **1**, but not in **2**. This ET reaction may at first seem unexpected as the reactants are linked via PhO^•^, but it is consistent with the observations and with the CSS being undetectable in **4**–**8**. The PCEnT process is of great interest, but population of the short-lived *[PhO=pyH] state also leads to rapid loss of CSS. Therefore, it is important to characterize both pathways of *[PhO=pyH] formation, either by PCEnT or by ET. The results show that the proposed competition between photoinduced proton-coupled energy vs electron transfer reactions gives very different excited state dynamics with even small changes in energetics induced by substituents or temperature. This competition is important for the development of photochemical systems with a function relying on a PCET or PCEnT, and it is interesting for its relationship with the more studied competition between noncoupled energy transfer vs electron transfer.

## Materials and Methods

Compounds were available from previous studies.^9^ Experimental methods, software, and instrumentation for variable temperature transient absorption (TA) experiments are as previously described.^12^ Briefly, the triads were dissolved in toluene (Merck, spectroscopic grade). Samples were prepared in a long-necked 2 mm × 10 mm quartz cuvette, and concentrations were adjusted to have an absorbance of ∼0.4 at the excitation wavelength (400 nm). Upon changing the temperature with a liquid-nitrogen-cooled cryostat, the sample was allowed to equilibrate for at least 1 h prior to data collection. The additional optical path length due to the cryostat resulted in a large initial artifact in the TA data. To account for this, spectra were chirp-corrected, and fitting was limited to times after ∼0.5–0.9 ps. Data were fit by both global analysis to a sequential model and target analysis (see the Supporting Information).

## Results

**Figure 3:**
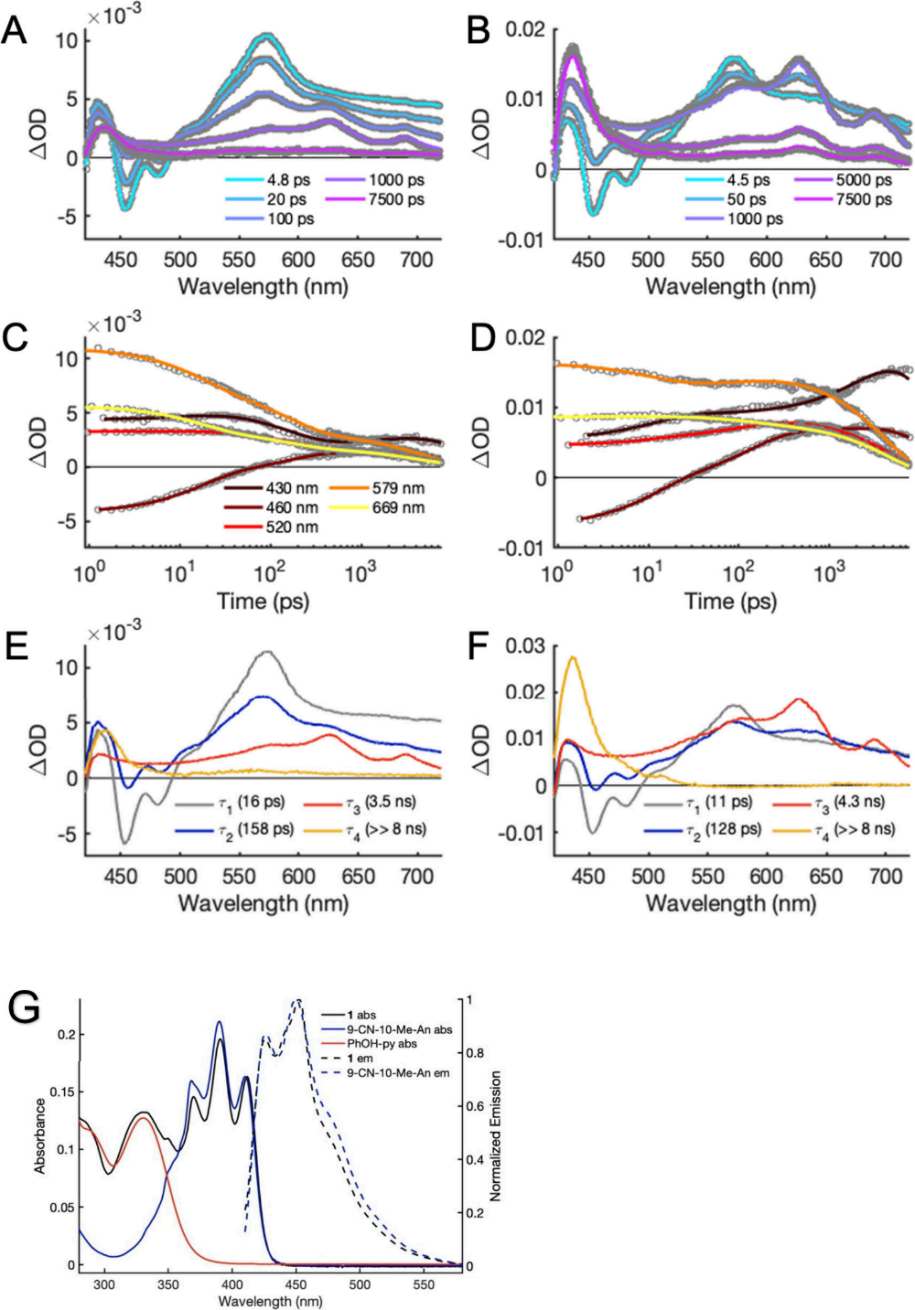
UV–vis TA spectroscopy data of **1** (A, C, E) and **2** (B, D, F) in toluene at 185 K and absorption spectra (solid) and fluorescence spectra with excitation at 400 nm (dashed) in room-temperature acetonitrile (G). (A, B) Spectral evolution at selected time points. (C, D) Kinetic traces at selected wavelengths. (E, F) Evolution associated spectra (EAS) from global fitting to a four-component sequential model. The EAS are the absorption spectra of the species corresponding to the given time constant. (G) Spectra for **1** (black), 9-CN-10-Me-anthracene (blue), and 2,2′-phenol-pyridine (red). Figure 3G is redrawn from ref (9). Copyright the American Association for the Advancement of Science, 2019.

Transient absorption (TA) experiments in toluene were conducted for **1** and **2** from 298 to 185 K. TA spectra at 185 K are shown in Figure 3 (for all other temperatures, see Supporting Information Figures S1–S28). The observed spectral features and their assignments at all temperatures are consistent with previously reported results in nitrile solvents and 298 K toluene.^9,12^ Light excitation forms ^1^*^^An that vibrationally relaxes within a few picoseconds (Figure 3A and B, spectra at ∼5 ps). The ^1^*^^An shows stimulated emission from 450 to 500 nm and a broad excited state absorbance above 500 nm, with a maximum at ∼575 nm. Over the next hundreds of picoseconds, the CSS formation is observed (Figure 3A and B, see spectra at ∼1000 ps). The CSS shows a narrow absorption centered at 425 nm (assigned to the PhO^•^ part of the triad) and a broad absorption from 475 to 700 nm with peaks at ∼625 and ∼685 nm (assigned to An^•–^).^12^ Finally, after a few nanoseconds, ^1^*^^An and the CSS have fully decayed and the TA spectra consist only of a narrow absorption centered at ∼435 nm which persists beyond the time window of the experiments (Figure 3A and B, see spectra at 7500 ps). The ∼435 nm band grows in on the time scale of CSS decay and is assigned to the ^3^*^^An based on spectral agreement. This suggests that some of the CR leads to formation of ^3^*^^An, consistent with our previous studies where ^3^*^^An was observed in toluene at room temperature and in dichloromethane^9^ but not in more polar solvents (butyronitrile, acetonitrile, and dimethylformamide).^9,12^ Direct formation via intersystem crossing from ^1^*^^An can be excluded because ^3^*^^An grows in on the time scale of CSS decay, not on the time scale of ^1^*^^An decay.

The TA spectra in toluene at all temperatures were globally fit with a sequential model with three lifetimes (time constants), τ_1_–τ_3,_ and an offset capturing the remaining signal at the end of the experiment (τ_4_ ≫ 8 ns). The resultant evolution associated spectra (EAS) at 185 K are shown in Figure 3E and F (for all other temperatures, see the Supporting Information). Target analysis was also performed to model two competing decay pathways for the CSS: inverted region CR to the GS and the formation of ^3^*^^An. The lifetimes reported in Table 1 are from global analysis and are consistent with results from target analysis. The state assignment corresponding to each lifetime is based on the TA spectral features of the components, as discussed elsewhere (Table 1).^12^ The state assignment is as follows: for **1** at all temperatures and for **2** at low temperatures (<240 K), the first component is assigned to the vibrationally “hot” ^1^*^^An and the second one to the thermally relaxed ^1^*^^An. The “hot” ^1^*^^An mainly undergoes thermal relaxation, whereas the thermally relaxed ^1^*^^An reacts to form the CSS. In the case of **1**, we propose that the ^1*^An also forms the *[PhO=pyH] state (*vide infra*). For **2** at high temperatures (240–298 K), the second and third components have similar EAS corresponding to the CSS. The second component was tentatively assigned to vibrational or structural relaxation^12^ as the EAS amplitudes and spectral features are similar; *i.e.*, there are only small TA signal changes represented by τ_2_ for this triad at 240 K and above. The main reactivity of ^1^*^^An in the triads is thus represented by τ_2_, except for **2** at 240–298 K where it is represented by τ_1_. The different assignment for **2** at high and low temperatures, respectively, explains why τ_1_ and τ_2_ for **2** are smaller at 220 K than at 240 K. For both **1** and **2** at all temperatures, the third component is assigned to the CSS and the fourth one to ^3^*^^An (τ ≫ 8 ns).

**Table 1:** Lifetimes^a^ from Global Fits to the TA Data and Corresponding State Assignments^b^

	Triad **1**			Triad **2**		
	τ_1_ (ps)	τ_2_ (ps)	τ_3_ (ps)	τ_1_ (ps)	τ_2_ (ps)	τ_3_ (ps)
Assignment/*T* (K)	^1^*^^An_hot_	^1^*^^An	CSS	^1^*^^An_hot_	^1^*^^An	CSS
185	16	158	3550	11	128	4320
200	13	108	3470	9	79	3850
220	13	77	2550	8	53	3790
240	11	56	1350	17	94^b^	2780
260	10	40	550	17	409^b^	2830
280	10	37	297	14	191^b^	2800
298	10	32	140	12	103^b^	2540

a Standard deviations of the lifetimes are ±5%.

b For **2** at high temperatures, the second lifetime component represents only very small amplitude changes (see text).

Despite structural and energetic similarities, **1** and **2** show significant differences in the relative TA amplitudes (ΔOD) for CSS and ^3^*^^An with respect to the initial amplitudes for ^1^*^^An (cf. Figure 3E and F). Estimated quantum yields for the formation of CSS (Φ_CSS_) and ^3^*^^An (Φ_T_) for **1** and **2** are given in Table 2. These are calculated using TA absorption amplitudes and extinction coefficients (ε) of the three species, ^1^*^^An, the CSS, and ^3^*^^An,^9,16−19^ according to eq 2 and eq 3, following the same procedure as that for data collected in toluene at 298 K.^12^ The uncertainty for the individual values is estimated to be ±33%, largely due to the uncertainty of the extinction coefficients. The relative values between temperatures and between the two triads are more accurate, with an estimated uncertainty of only ca. ±10% (see Section 3 of the Supporting Information for details).
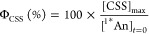
2
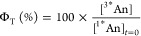
3

**Table 2:** Maximum Observed Yields^a^ of the Transient CSS and ^3^*^^An with Respect to the ^1^*^^An_hot_ for **1** and **2** in Toluene

	Triad **1**		Triad **2**	
*T* (K)	Φ_CSS_ (%)	Φ_T_ (%)	Φ_CSS_ (%)	Φ_T_ (%)
185	34	3.8	81	12
200	33	3.7	84	11
220	33	2.8	88	13
240	32	2.7	89	12
260	39	1.1	80	10
280	29	0.5	92	8.5
298	26	0.5	90	6.8

a The estimated uncertainty for individual values is ±33%; the relative uncertainty between values is ±10%. See Section 3 of the Supporting Information for details.

Comparing the two triads, Φ_CSS_ values for **1** are substantially smaller than those for **2** over the studied temperature range, with Φ_CSS_ for **1** varying from 26 to 35% vs 81 to 90% for **2**. Φ_CSS_ values for **1** and **2** seem to show opposite trends with temperature, though this is within the relative error of the measurements (±10%). The much lower Φ_CSS_ for **1** cannot be explained by CS being slow compared to direct deactivation to the ground state, as the lifetime of ^1^*^^An in **1** was at least 100-fold shorter than that for unquenched anthracene (τ ≈ 17 ns).^12^ Instead, the much lower Φ_CSS_ for **1** shows that an additional excited state reaction is active, and this path possibly contributes to a smaller extent in **2**. We propose that this additional reaction is PCEnT from ^1^*^^An to form *[PhO=pyH], as discussed below. The differences in Φ_CSS_ for **1** and **2** in toluene contrast with the data in butyronitrile, where the estimated Φ_CSS_ is >80% for both triads over a similar temperature range.^12^

For both triads, ^3^*^^An is formed on the time scale of the CR, long after the disappearance of ^1^*^^An. The ^3^*^^An state is formed by charge recombination of the singlet CSS (^1^CSS), either directly to the ^3^*^^An state or via ISC to the triplet CSS (^3^CSS), followed by CR on the triplet surface; see the Discussion below. For **1** and **2**, the Φ_T_ values are significantly smaller than their respective Φ_CSS_, showing that most CR still occurs via a singlet pathway directly to the ground state. From the values of the CSS lifetime and Φ_T_ we calculate a rate constant for triplet formation of *k*_T_ ≈ 3 × 10^7^ s^–1^ at all temperatures. As the temperature increases, Φ_T_ decreases for both triads, approximately following the decrease in the CSS lifetime.

**Figure 4:**
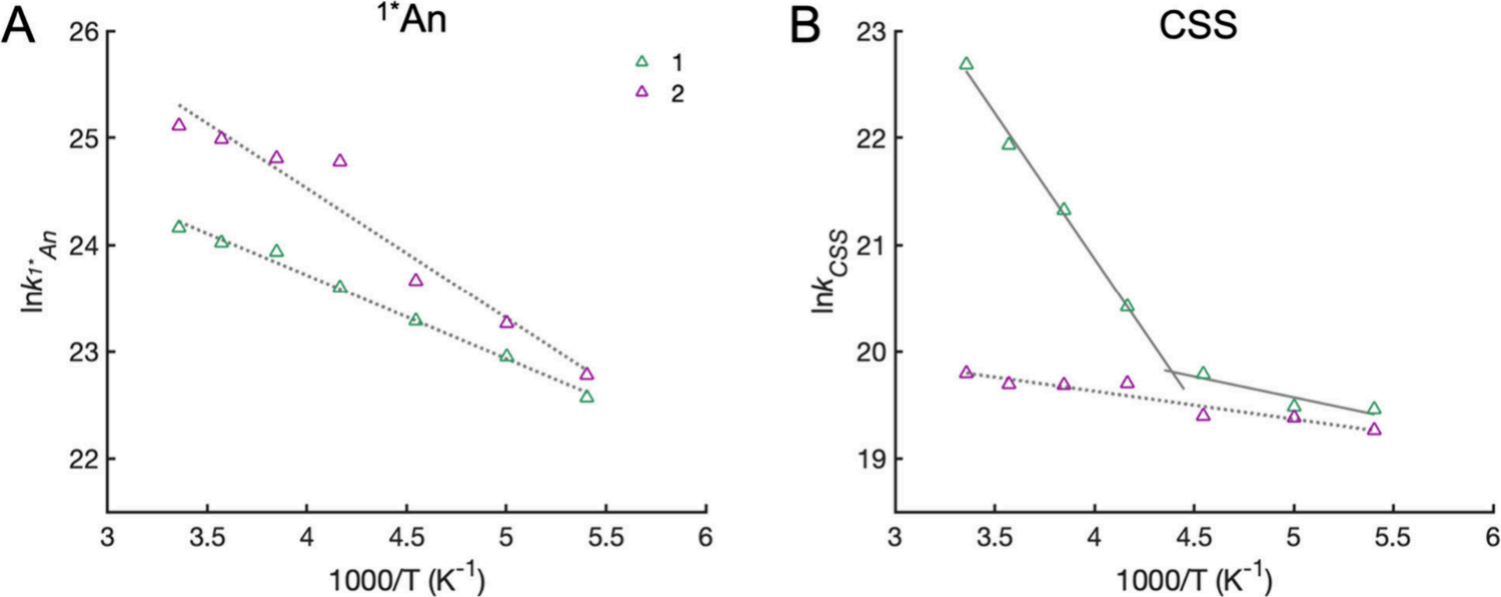
Arrhenius plots for **1** (green) and **2** (purple) in toluene. The analysis was performed using the overall rates of decay for ^1^*^^An (A) and CSS (B), which include multiple competing reaction pathways.

The ^1^*^^An lifetimes for **1** and **2** show a similar temperature dependence, but the CSS lifetimes do not (Figure 4). For ^1^*^^An, the lifetime increases upon cooling from 298 to 185 K, by a factor of 5–10, similar to results in butyronitrile.^12^ In contrast, the CSS lifetime for **1** shows an ∼25-fold increase in the same temperature range, whereas for **2** it shows a mere 1.7-fold increase (Table 1). The temperature dependence for **2** is similar to that in butyronitrile, where the CSS lifetimes increase 2-fold for both **1** and **2**.^12^

The different temperature dependencies are shown in the Arrhenius plots in Figure 4. Rate constants for deactivation of ^1^*^^An and CSS were calculated using the values in Table 2 and the relationships  = 1/τ_2_ and *k*_CSS_ = 1/τ_3_ for **1** at all temperatures and for **2** at low (<240 K) temperatures. For **2** at high temperatures, the relationship  = 1/τ_1_ was used instead, following the time constant assignment discussed above. The data for **1** fit well to the Arrhenius equation (*R*^2^ = 0.99), while a slightly poorer fit is obtained for **2** (*R*^2^ = 0.94). This may be because the data for **2** at 298 to 240 K is for the reaction from a vibrationally excited ^1^*^^An that would be expected to react faster than thermally relaxed states. The activation energies calculated for  (∼70 and ∼100 meV for **1** and **2**, respectively) are similar to those observed for the rate constants of CS for both triads in butyronitrile (∼70 meV).^12^

In contrast to the above similarities, the Arrhenius plots for *k*_CSS_ are starkly different for **1** vs **2** (Figure 4B). **2** shows a weak temperature dependence over the entire range with *E*_a_ = 23 ± 3 meV. At low temperatures, **1** shows a similarly weak dependence (*E*_a_ = 34 ± 15 meV), but above 220 K, the dependence is much stronger, with 1 order of magnitude greater activation energy (*E*_a_ = 240 ± 10 meV). This suggests that **1** at low temperatures follows the same mechanism as that in **2** over the entire temperature range, but an additional CR reaction pathway is accessible for **1** at *T* > 220 K. The latter is an important result that we discuss below.

Despite the inherent shortcomings of the Arrhenius model, we did not pursue more complex models (*e.g.*, using eq 1 with multiple vibronic transitions or the Marcus electron transfer expression accounting for Δ*G°* and λ temperature variations, as in our previous work in butyronitrile^12^). These models were not accessible because of the difficulties in estimating Δ*G°* and λ in nonpolar solvents. The Arrhenius analysis still provides qualitative insights into the differences between the two triads, and the same triad in different solvents.

## Discussion

### Inverted Region Behavior for CR

Our previously reported TA results for **1** and **2** in toluene at room temperature^12^ (*ε*_s_ = 2.38 at 296 K^20^) indicated significantly different dynamics than those observed in more polar solvents.^9,12^ First, the overall CSS lifetime of **2** was 2.5 ns. Outside of toluene, the longest CSS lifetime we had observed was 755 ps for **1** in dichloromethane at room temperature (*ε*_s_ = 8.93 at 298 K^20^).^9^ The long lifetime in toluene is consistent with the large solvent effect predicted for inverted region CR,^21,22^ but the magnitude of the effect is notable: a factor of 3 longer lifetime vs dichloromethane. In a nonpolar solvent, the zwitterionic CSS is expected to be highly destabilized, thereby decreasing the driving force for CS and increasing the driving force for CR. Following Marcus theory,^23,24^ these changes in driving force are predicted to result in slower rates for both normal region CS and inverted CR. In addition to the effect on driving force, however, lowering the solvent polarity will also decrease the reaction reorganization energy. Reducing the reorganization energy is predicted to accelerate rates for a normal reaction while slowing rates for an inverted reaction. Taking into consideration both of the expected trends for driving force and reorganization energy, the solvent effect for CS is expected to be smaller, and for CR, it is expected to be larger. Consistent with these predictions, the low polarity of toluene pushes CR further into the inverted region resulting in nanosecond lifetimes of the CSS, whereas CS at room temperature is similarly fast as in the more polar solvents (e.g., 10 ps for **2** vs 5 ps in butyronitrile^12^).

The observed time constant for the decay of the CSS for **2** is longer than 2.5 ns at all temperatures. The nanosecond lifetime for CSS suggests that the inverted effect could be important in energy conversion processes. During photosynthesis, photon absorption induces charge transfer and the formation of CSSs.^25^ These CSSs are highly reactive and susceptible to undergoing unproductive CR reactions, thus wasting energy and resulting in low quantum efficiencies. For a two-level photochemical system modeled by Ross in 1967,^26^ it was determined that the light-to-chemical power conversion can be maximized by reducing the rate of reactions that are not coupled to the power conversion process. The rates of such unproductive reactions can be slowed if their driving force lies in the Marcus inverted region. This inverted effect has been proposed to explain the near unity quantum yields for long-lived charge separation in photosynthesis.^27^ Also in synthetic systems, this may be a potential solution to selectively slow down unproductive CR reactions and allow the desired, fuel-producing reactions to dominate.^27−29^ In this work, we have taken advantage of CR being inverted in order to achieve CSS lifetimes on the order of a few nanoseconds. This is significant, as the CSS persists long enough that it should be able to participate in bimolecular reactions in solution.

### PCET vs PCEnT

The results in toluene show that both the reactions of ^1^*^^An and the CSS involve parallel reactions with CS and CR, respectively. The reaction of ^1^*^^An results in near-quantitative formation of CSS in **2**; the quantum yield at 298 K is within experimental error equal to 100%, and the apparent slight decrease at lower temperatures can be an effect of different estimates of initial hot vs thermalized ^1^*^^An signal magnitudes at the different temperatures. For **1**, instead, Φ_CSS_ is much lower, and the question is what additional reaction is deactivating ^1^*^^An. Our previous studies showed that ^1^*^^An triad states can undergo the newly discovered PCEnT reaction to form *[PhO=pyH], which for **1** occurs with a time constant of ∼69 ps in 77 K butyronitrile glass.^13^ It is reasonable to assume that PCEnT occurs on at least a similar time scale in room-temperature toluene in the present experiments, which would make it competitive in deactivating the ^1^*^^An state (τ_2_ ≈ 30–160 ps). Therefore, we propose that PCEnT occurs also in fluid toluene solution of **1**, and possibly to some extent also of **2**, and that this is the additional ^1^*^^An deactivation pathway leading to relatively low values of Φ_CSS_ for **1**. In fluid solution, the *[PhO=pyH] state is very short-lived and cannot be directly observed, since it rapidly reaches a conical intersection and deactivates to the ground state.^30,31^ Therefore, no TA signal or fluorescence is expected from the *[PhO=pyH] intermediate. The modest variation in Φ_CSS_ with temperature shows that PCET and PCEnT have similar activation energies in **1**. The smaller, possibly even negligible, contribution of PCEnT in **2** can be explained by the larger rate constant for PCET compared to that in **1** and that the *[PhO=pyH] state lies higher in energy in **2** (ca. 260 meV larger adiabatic excitation energy from gas-phase calculations).^13^ We used the values of Φ_CSS_ and ^1^*^^An lifetime to calculate the rate constants for *k*_CS_ and *k*_PCEnT_, assuming that ^1^*^^An is significantly deactivated only by CS and PCEnT, such that *k*_CS_ = Φ_CSS_/τ_2_ and *k*_PCEnT_ = (1 – Φ_CSS_)/τ_2_ (with τ_1_ instead of τ_2_ for **2** and *T* ≥ 240 K; see Table 3). An Arrhenius analysis using these values for *k*_CS_ resulted in activation energies for **1** and **2** that are within error of the fits discussed in the Results section (see Section 4 of the Supporting Information).

**Table 3:** Extracted Rate Constants for CS, PCEnT, and CR in **1** and **2**

	Triad **1**				Triad **2**		
*T*/K	*k*_CS_ (10^9^ s^–1^)^a^	*k*_PCEnT_ (10^9^ s^–1^)^b^	*k*_CR_ (10^9^ s^–1^)^c^	*k*_ET_ (10^9^ s^–1^)^c^	*k*_CS_ (10^9^ s^–1^)^a^	*k*_PCEnT_ (10^9^ s^–1^)^b^	*k*_CR_ (10^9^ s^–1^)^d^
185	2.1	4.2	0.17	0.02	6.3	1.5	0.20
200	3.1	6.2	0.21	0.06	11	2.1	0.23
220	4.2	8.7	0.26	0.23	17	2.2	0.23
240	5.7	12	0.31	0.67	51	6.4	0.31
260	9.6	15	0.37	1.7	47	12	0.31
280	7.9	19	0.42	3.8	65	5.9	0.32
298	8.1	23	0.46	6.9	73	7.8	0.36

a Calculated from *k*_CS_ = Φ_CSS_/τ_2_ (from *k*_CS_ = Φ_CSS_/τ_1_ for **2** at *T* ≥ 240 K).

b Calculated from *k*_PCEnT_ = (1 – Φ_CSS_)/τ_2_ (from *k*_CS_ = (1 – Φ_CSS_)/τ_1_ for **2** at *T* ≥ 240 K).

c Calculated from the Arrhenius parameters, correcting for the small amount of triplet formation (*k*_T_ ≈ 3 × 10^7^ s^–1^); see Section 4 of the Supporting Information.

d Calculated from .

An exact mechanistic understanding of PCEnT remains to be developed—although an elaborate theory has recently been published^32^—but a few comments are appropriate to include here. In the original paper,^13^ we could on energetic grounds exclude stepwise reactions via the excited *enol form or via the ground state keto tautomer. In that study, in rigid solvent glass at 77 K, we could also exclude formation of the *[PhO=pyH] state via initial formation of the CSS; see the Discussion below, however, regarding the situation in fluid toluene. It was therefore clear that PT and EnT occurred in a concerted reaction. Regarding the nature of the coupling, it is interesting to compare with PCET reactions, where the charge redistribution between donor and acceptor due to the individual ET and PT contributions are strongly coupled. In contrast, PCEnT gives no charge redistribution between the energy donor and acceptor in the triads, although there is some redistribution *within* the phenol-pyridine unit. The tautomerization of the latter unit nevertheless changes its excited state energy; it was therefore suggested that thermal fluctuations of the internal structure and solvent bring the excitation energies of the anthracene and phenol-pyridine units into resonance, so that PCEnT can occur. Finally, the electronic coupling was proposed to be dominated by exchange or other short-range mechanisms, as the donor-acceptor distance was small, and there was no observable spectral overlap of anthracene fluorescence and phenol-pyridine absorption (cf. Figure 3G). Further work is in progress to better understand the PCEnT reactions.

### CSS Deactivation

Next, we discuss the reactions from the CSS. The activation energies for CR in **2** and for **1** at *T* > 220 K are very similar to the apparent activation energy in butyronitrile (23 meV),^12^ which may seem to contradict the expected effect due to CR being deeper into the inverted region in toluene. This can be rationalized, however, by noting that the PCET reaction involves several vibronic transitions (μ → ν in eq 1) and that the dominating transitions are different in the different solvents and at different temperatures, as was discussed before.^12^ In the inverted region, CR to higher vibrational states of the products lowers the barrier because Δ*G*°_μν_ becomes less negative. On the other hand, the vibrational wave function overlap becomes smaller with higher ν-values.^9,33^ To compensate for a large barrier in the inverted region, the reaction may thus find a compromise by reacting via higher vibrational states at the expense of a poorer wave function overlap. The effective temperature dependence can therefore be quite parallel in the two solvents, even if the reaction is more inverted and is slower in toluene, as observed.

The Arrhenius plot for the CR in **1** is biphasic, which strongly suggests an additional pathway from the CSS at high temperatures. No new features appear in the TA spectra, indicating that this additional state is spectroscopically dark under our experimental conditions or deactivates to the ground state faster than it is formed. Since also the ^3^*^^An yield in **1** decreases strongly at higher temperatures, much more than the drop in CSS yield, CSS deactivation at high temperatures is not due to an increased importance of the triplet CR pathway. Taken together, these findings indicate an additional reaction from the CSS in **1** at higher temperatures that competes with CR to the ground state and with ^3^*^^An formation.

Although estimating driving forces in nonpolar media is challenging, it is clear that the CSS is destabilized in toluene compared to in polar solvents, and its energy should approach that of the *[PhO=pyH] state. Therefore, we propose that, for **1**, *[PhO=pyH] could form from the CSS, by electron transfer (ET) from the An^•–^ to the pyH^+^ unit leading to the excited keto tautomer of the phenol-pyridine. The *[PhO=pyH] state is calculated to lie 260 meV higher in **2** than in **1** (gas phase value; see above) which could explain why this pathway is observed only for **1**. This reaction pathway is consistent with all other direct observations and could account for the high *E*_a_ at high temperatures for **1**. The population of *[PhO=pyH] from both ^1^*^^An and CSS is also consistent with the trends in CSS and ^3^*^^An formation yields between the triads. A PCEnT transition from ^1^*^^An to *[PhO=pyH] could account for the lower CSS yields for **1** vs **2**. The proposed ET from CSS to *[PhO=pyH] could explain the much faster CR above 220 K for **1** and its lower yields of ^3^*^^An. The calculated rate constants for the different CSS deactivation pathways are given in Table 3 (see Section 4 of the Supporting Information for details).

The CSS-to-*[PhO=pyH] ET reaction may at first seem surprising, as it is formally the transfer of an electron from the An^•–^ unit to the pyH^+^, via the neighboring PhO^•^, instead of direct transfer to the PhO^•^ unit itself (CR). However, it should first be noted that the resulting *[PhO=PyH] state is delocalized over that unit, so that electronic coupling with anthracene states can be large. Second, CR is in the inverted region with small proton vibrational overlap for low-barrier transitions (see above), whereas ET to form *[PhO=pyH] has a much smaller driving force as the product is electronically excited, and it is not coupled to PT. We thus propose that the preference for ET at *T* ≥ 240 K is yet another manifestation of the inverted region effect for CPET.

**Figure 5:**
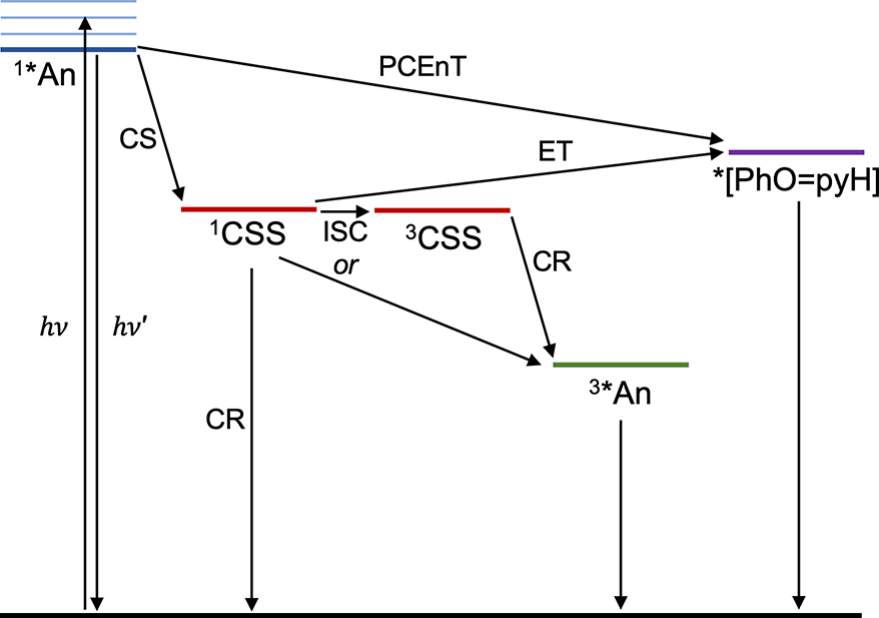
Jablonski diagram showing the reactions from ^1^*^^An proposed for **1** in toluene; see text.

The formation of ^3^*^^An from the CSS can in principle occur in a direct step, or via ISC from ^1^CSS to ^3^CSS followed by charge recombination to ^3^*^^An.^34−36^ Zeeman and hyperfine interactions can cause ^1^CSS–^3^CSS ISC in weakly coupled radical pairs (Δ*E*_ST_ ≈ 1 cm^–1^ or smaller), but based on comparisons with other closely linked donor-acceptor systems in the literature, our radical pairs may be too strongly coupled for that.^37,38^ Spin-orbit coupling is usually not expected to be effective for ^1^CSS and ^3^CSS that share the same orbital parentage but has been calculated to be effective at distances up to a few Angstrom for some radical pairs.^39^ Spin-orbit coupling in a direct ^1^CSS-to-^3^*^^An step may also be feasible, favored by configurations where the PhO=PyH ring plane is rotated to be at a 90° angle with respect to that of the An (with a strong change in orbital angular momentum upon electron transfer).^34,37^ A clear determination of the mechanism for ^3^*^^An formation from ^1^CSS would require time-resolved EPR experiments, but ^3^*^^An formation is a minor pathway and is not the focus of the present study. We finally note that formation of ^3^*^^[PhO=PyH] cannot be excluded based on the data. In Figure 5 below, we therefore represent the *[PhO=PyH] state without a spin label, to open for this possibility.

### Jablonski Diagram for the Triad Photoreactions

The proposed transitions from CSS for **1** in toluene are summarized in Figure 5. Light excitation results in a vibrationally excited ^1^*^^An (indicated by the multiple thin blue lines), which rapidly thermalizes (to the thick blue line). The thermalized ^1^*^^An decays via two competing pathways: PCEnT to form *[PhO=pyH] and CS to form the CSS. The singlet ^1^CSS can undergo either CR to the ground state, ET to *[PhO=pyH], or CR to form ^3^*^^An (possibly via ^3^CSS formation) that has a lifetime ≫8 ns. *[PhO=pyH] deactivates rapidly in fluid solution via a conical intersection, as established in the ESIPT literature,^30,40^ to reform the enol form ground state. The energy of the CSS varies with the solvent and temperature and in 77 K glass is estimated to lie above the ^1^*^^An state.^13^

## Conclusions

Variable temperature TA measurements of the photoinduced reactions of An-PhOH-py triads **1** and **2** in toluene have revealed several decay pathways. The low polarity of the toluene seems to destabilize the charge separated state (CSS) sufficiently that PCEnT, to form the excited *[PhO=pyH] tautomer from the initially excited ^1^*^^An state, becomes competitive with PCET. Thus, PCEnT is suggested to occur not only in rigid media^13^ but also in fluid solution, at least in **1** and possibly also to some extent in **2**. The lower energy of the *[PhO=pyH] state in **1** than in **2** is consistent with PCEnT being more pronounced in **1**.

The results also suggest a new pathway for deactivating the CSS of **1** at *T* ≥ 240 K, and we propose this to be via formation of the same *[PhO=pyH] state. This reaction would formally be an ET from the An^•–^ to the pyH^+^ unit, forming the excited *[PhO=pyH] state while avoiding charge recombination (CR) with the intervening PhO* back to the ground state. For **2**, the *[PhO=pyH] state lies significantly higher in energy than that for **1**, which can explain why the same reaction is not observed in this triad. For triads **4**–**8** investigated before, the driving force for *[PhO=pyH] formation is instead larger, which would lead to an even faster CSS decay than that in **1**. The fact that the CSS could not be observed in **4**–**8** after quenching of ^1^*^^An in solution^9^ can thus be explained by either rapid ET from the CSS to form *[PhO=pyH], so that CSS never accumulates to a detectable level, or a faster PCEnT from ^1^*^^An that outcompetes PCET, or a combination of both.

Our results suggest more general involvement of the newly identified PCEnT reaction, including fluid solution conditions. They also suggest multiple ways of forming the excited state keto form of a classic ESIPT compound that otherwise is only formed by its direct excitation and ESIPT. Both direct PCEnT and ET from the CSS form the *[PhO=pyH] state by initial ^1^*^^An excitation. Thus, this state can be formed in two different ways by excitation of the anthracene unit, which is with much lower excitation energy than with direct excitation of the PhOH-py fragment.

The inverted region effect on CR for this PCET reaction is further extended in this study, giving a CSS lifetime in **2** of up to 2.5 ns at room temperature. This is long enough to undergo bimolecular reactions, and this is therefore an exciting step in the efforts to exploit light-induced PCET reactions for, e.g., solar energy conversion and photoredox catalysis. The results show that a long-lived CSS in the inverted region can indeed be obtained but also points to additional side reactions related to its proton-coupled nature that are important to consider.

## Supporting Information Available

Transient absorption data and fitted time constants, details and results of target analysis, and Arrhenius analysis with extracted rates (PDF)
