# Correction to “Evidence for Competing Proton-Coupled Reaction Pathways of Molecular Triads in a Low-Polarity Solvent”

**DOI:** 10.1021/acs.jpca.5c06487

**Published:** 2025-10-06

**Authors:** Laura F. Cotter, Giovanny A. Parada, Rohit Bhide, Belinda Pettersson Rimgard, James M. Mayer, Leif Hammarström

One of the grant numbers in the Acknowledgments was incorrect. The correct section is given below.

