# Supplementary material for: Evidence for Competing Proton-Coupled Reaction Pathways of Molecular Triads in a Low-Polarity Solvent
Source: J Phys Chem A. 2025 Feb 6;129(7):1792–800. doi: 10.1021/acs.jpca.4c05734 (PMC11848912; doi:10.1021/acs.jpca.4c05734)
Supplement: Supplementary file 1 — jp4c05734_si_001.pdf [file jp4c05734_si_001.pdf]

Supporting Information for

Evidence for Competing Proton-Coupled Reaction Pathways of Molecular Triads in a Low-polarity Solvent

Laura F. Cotter,[1] Giovanny A. Parada,[1,2] Rohit Bhide,[1] Belinda Pettersson Rimgard,[3] James M. Mayer,[1*] Leif Hammarström[3*]

[1] Department of Chemistry, Yale University, New Haven, Connecticut, 06520, USA

[2] The College of New Jersey, Department of Chemistry, Ewing, New Jersey 08628, USA

[3] Department of Chemistry – Ångström Laboratory, Uppsala University, Box 523, SE75120 Uppsala, Sweden

[*]Correspondence to: james.mayer@yale.edu, leif.hammarstrom@kemi.uu.se

Contents

Contents *S1*

1 Femtosecond UV-vis Transient Absorption Spectroscopy in Toluene S2

1.1 *Triad 1 Time Traces, Spectra, and EAS* *S2*

1.2 *Triad **2** Time Traces, Spectra, and EAS* *S7*

1.3 *Triad **3** Time Traces, Spectra, and EAS* *S11*

2 Formation Yields, Branching Ratios, and Target Analysis S12

3 Arrhenius Analysis with Extracted Individual Rate Constants S13

1 Femtosecond UV-vis Transient Absorption Spectroscopy in Toluene

1.1 Triad 1 Time Traces, Spectra, and EAS

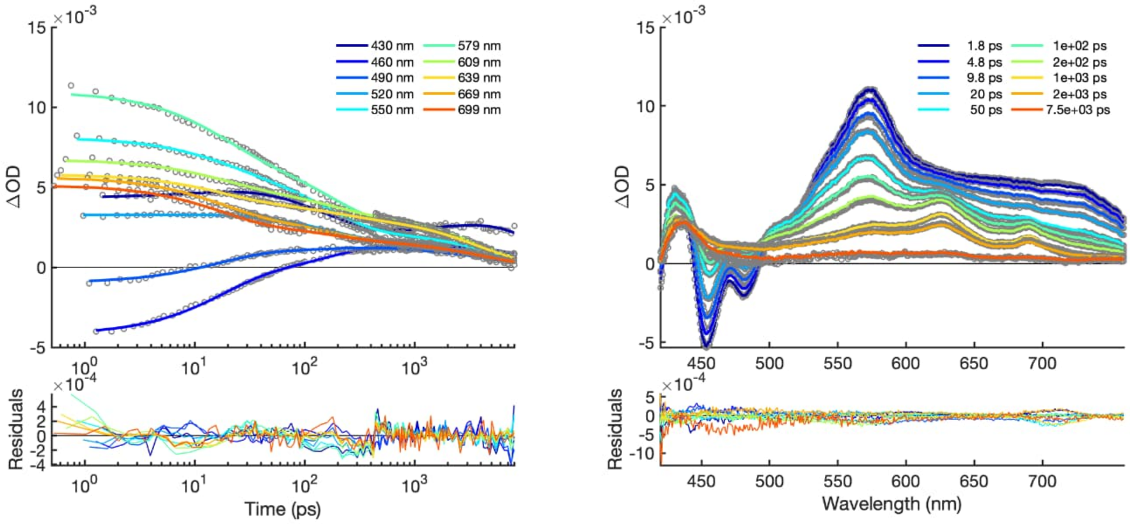

Figure S1. UV-vis TA spectroscopy of **1** in toluene at 185 K at selected wavelengths (left) and selected times (right). Time constants obtained from global analysis are τ_1 = 16 ps, τ_2 = 158 ps, τ_3 = 3.55 ns, and τ_4 >> 8 ns.

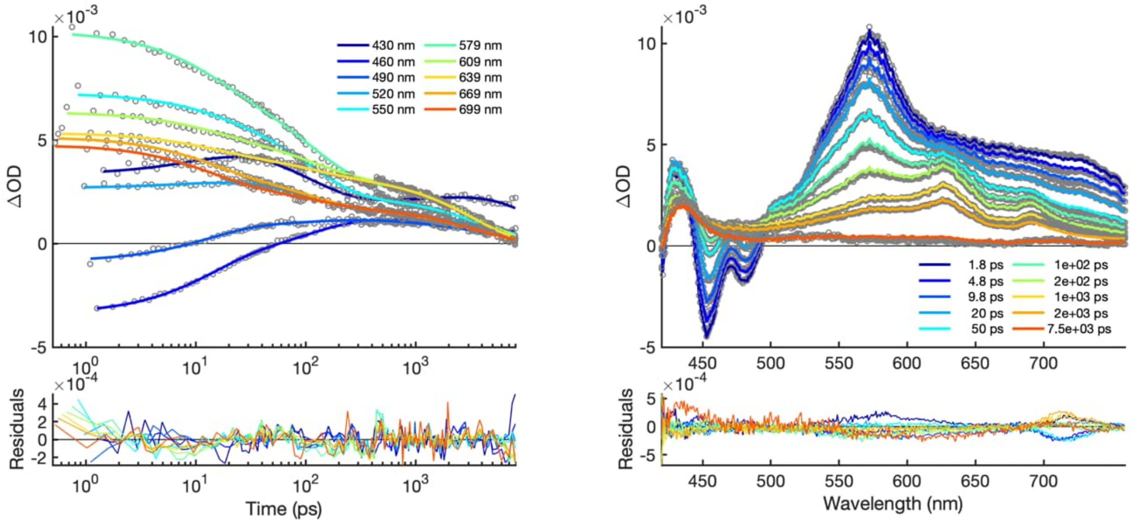

Figure S2. UV-vis TA spectroscopy of **1** in toluene at 200 K at selected wavelengths (left) and selected times (right). Time constants obtained from global analysis are τ_1 = 13 ps, τ_2 = 108 ps, τ_3 = 3.47 ns, and τ_4 >> 8 ns.

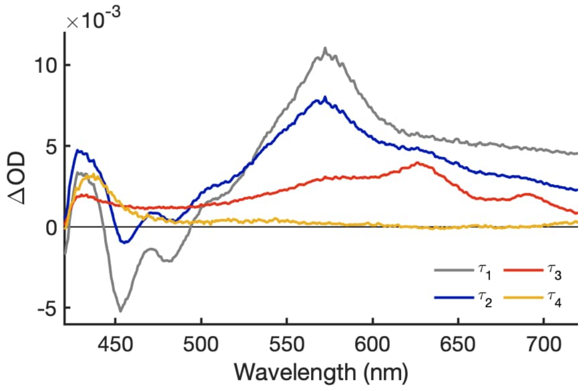

Figure S3. EAS obtained from global analysis for **1** in toluene at 200 K.

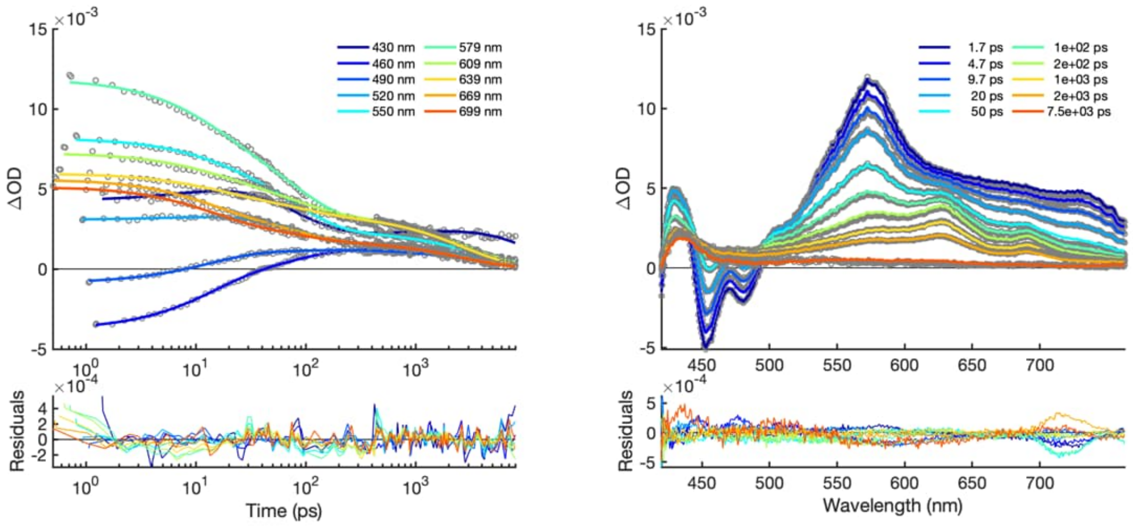

Figure S4. UV-vis TA spectroscopy of **1** in toluene at 220 K at selected wavelengths (left) and selected times (right). Time constants obtained from global analysis are τ_1 = 13 ps, τ_2 = 77 ps, τ_3 = 2.55 ns, and τ_4 >> 8 ns.

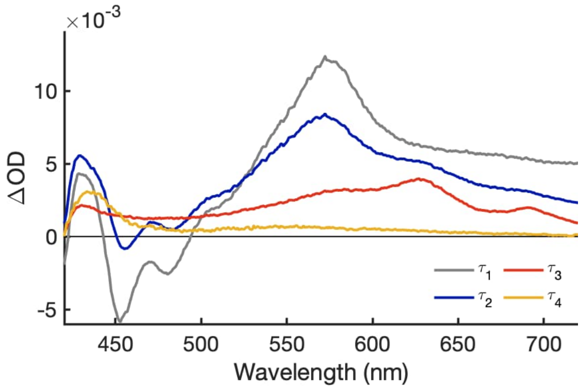

Figure S5. EAS obtained from global analysis for **1** in toluene at 220 K.

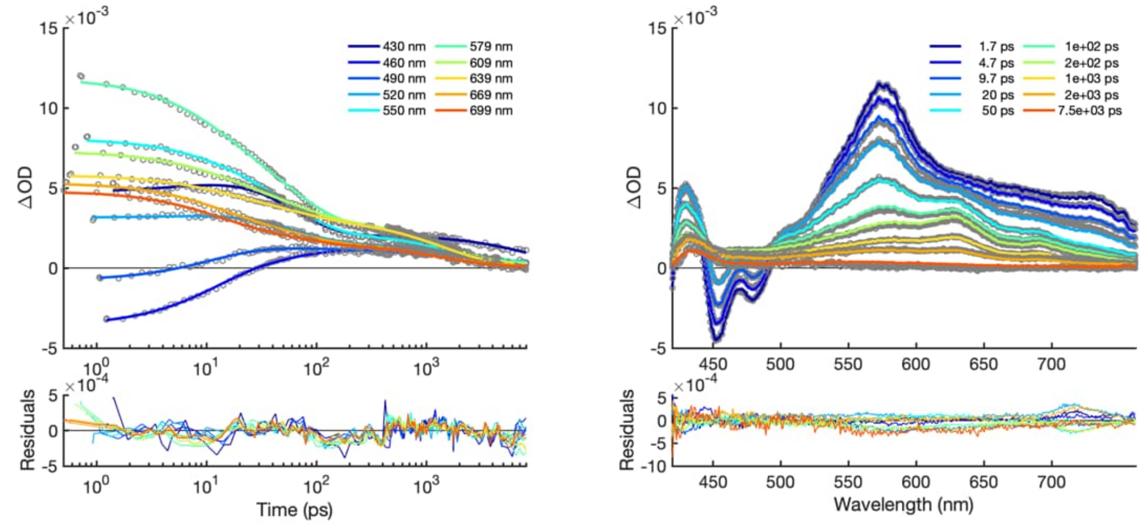

Figure S6. UV-vis TA spectroscopy of **1** in toluene at 240 K at selected wavelengths (left) and selected times (right). Time constants obtained from global analysis are τ_1 = 11 ps, τ_2 = 56 ps, τ_3 = 1.35 ns, and τ_4 >> 8 ns.

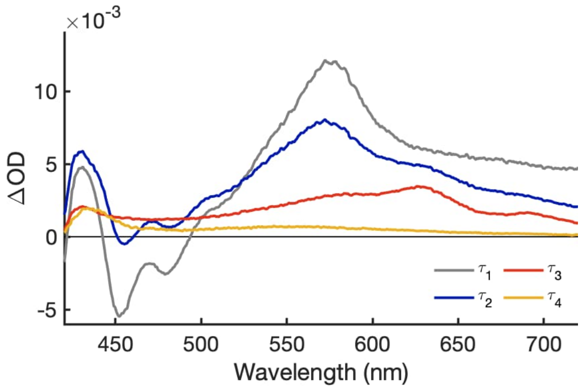

Figure S7. EAS obtained from global analysis for **1** in toluene at 240 K.

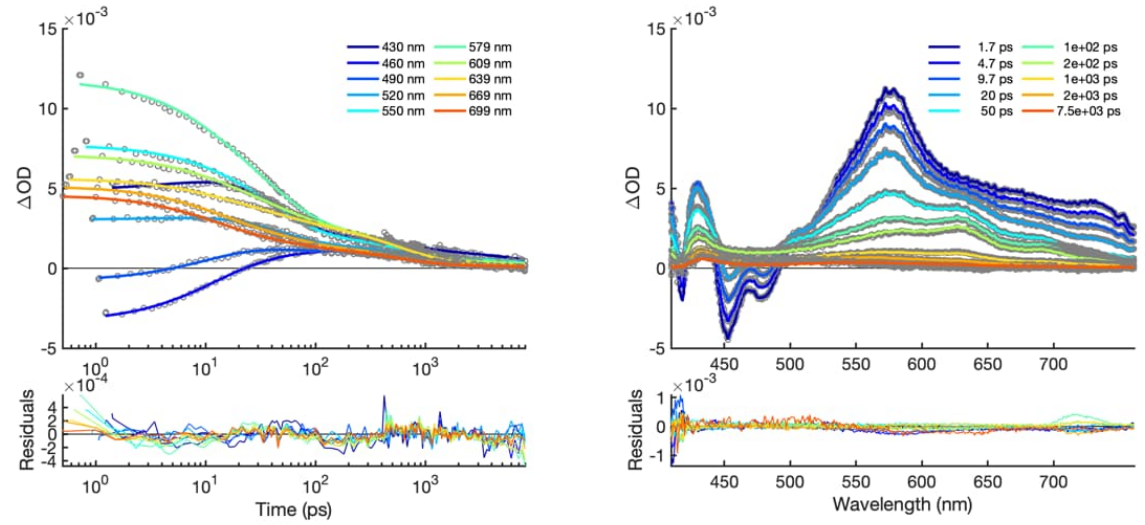

Figure S8. UV-vis TA spectroscopy of **1** in toluene at 260 K at selected wavelengths (left) and selected times (right). Time constants obtained from global analysis are τ_1 = 10 ps, τ_2 = 40 ps, τ_3 = 550 ps, and τ_4 >> 8 ns.

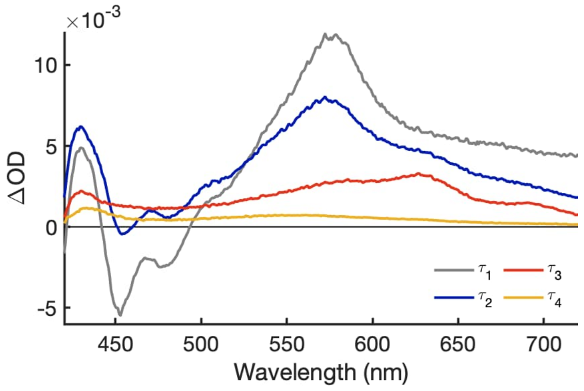

Figure S9. EAS obtained from global analysis for **1** in toluene at 260 K.

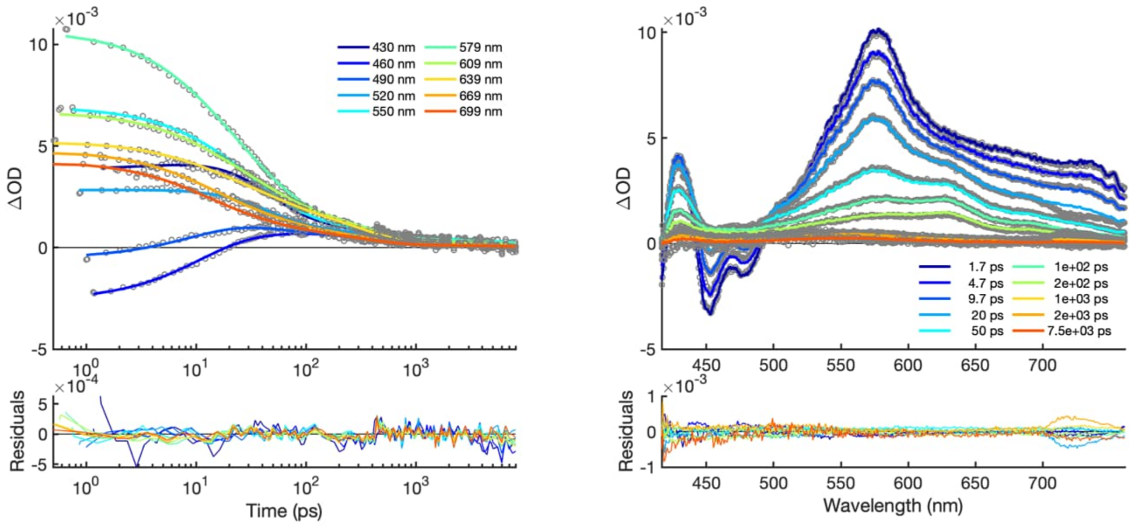

Figure S10. UV-vis TA spectroscopy of **1** in toluene at 280 K at selected wavelengths (left) and selected times (right). Time constants obtained from global analysis are τ_1 = 10 ps, τ_2 = 37 ps, τ_3 = 297 ps, and τ_4 >> 8 ns.

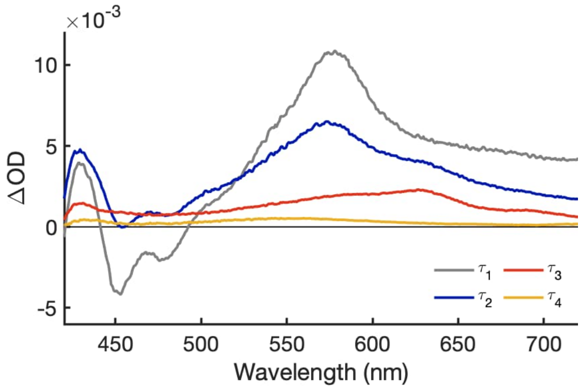

Figure S11. EAS obtained from global analysis for **1** in toluene at 280 K.

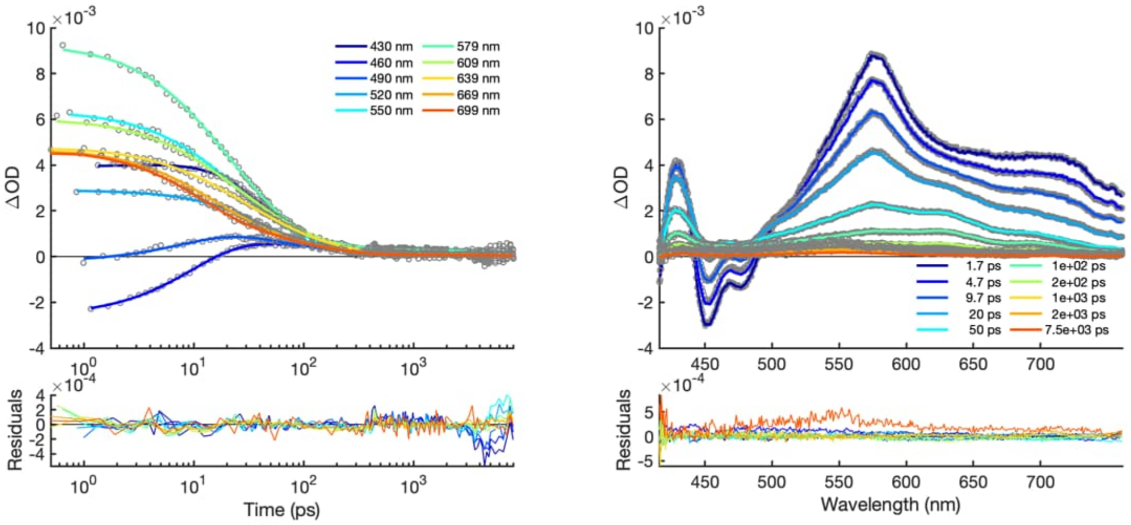

Figure S12. UV-vis TA spectroscopy of **1** in toluene at 298 K at selected wavelengths (left) and selected times (right). Time constants obtained from global analysis are τ_1 = 10 ps, τ_2 = 32 ps, τ_3 = 140 ps, and τ_4 >> 8 ns. Note that this data was also reported in reference [1].

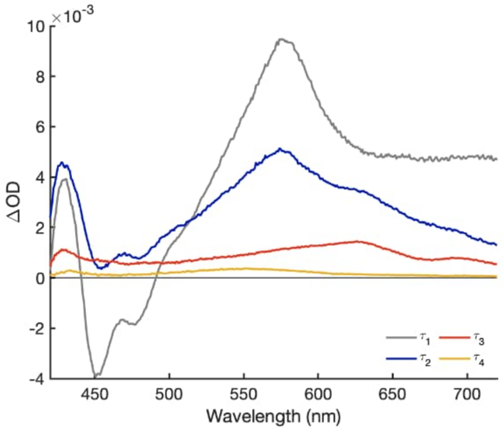

Figure S13. EAS obtained from global analysis for **1** in toluene at 298 K.

1.2 Triad **2** Time Traces, Spectra, and EAS

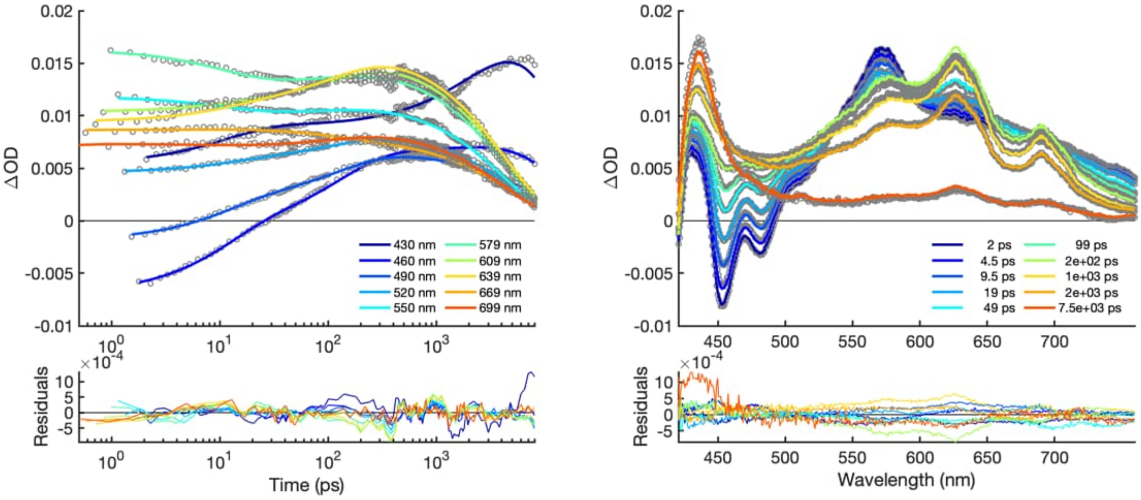

Figure S14. UV-vis TA spectroscopy of **2** in toluene at 185 K at selected wavelengths (left) and selected times (right). Time constants obtained from global analysis are τ_1 = 11 ps, τ_2 = 128 ps, τ_3 = 4.32 ns, and τ_4 >> 8 ns.

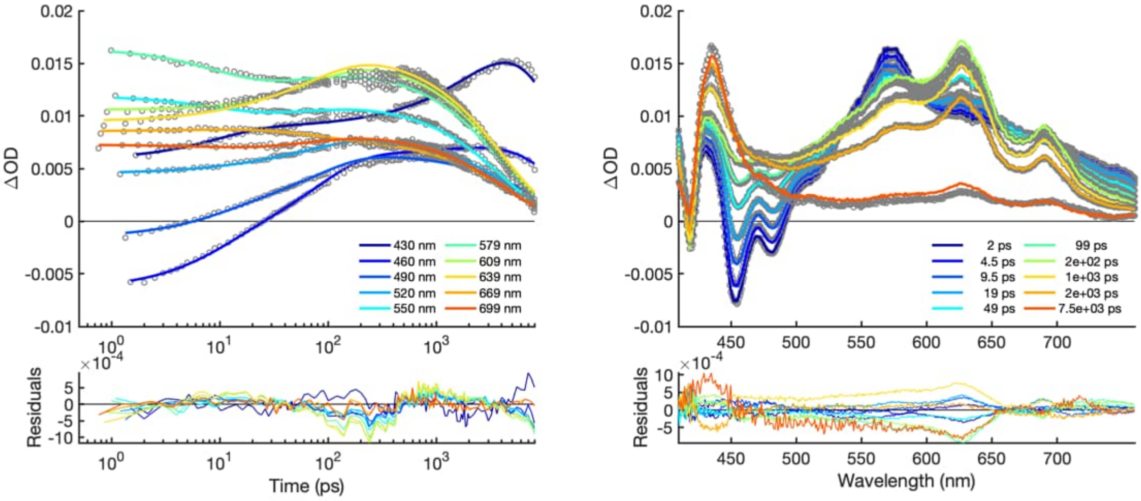

Figure S15. UV-vis TA spectroscopy of **2** in toluene at 200 K at selected wavelengths (left) and selected times (right). Time constants obtained from global analysis are τ_1 = 9 ps, τ_2 = 79 ps, τ_3 = 3.85 ns, and τ_4 >> 8 ns.

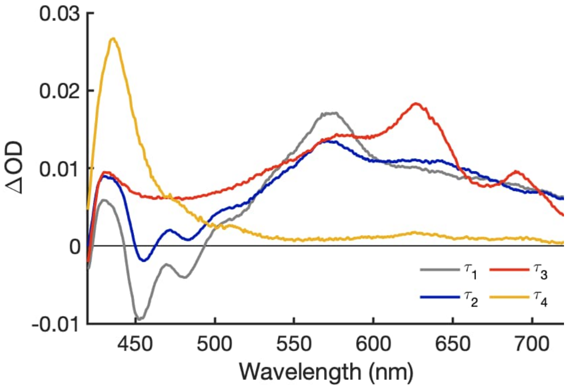

Figure S16. EAS obtained from global analysis for **2** in toluene at 200 K.

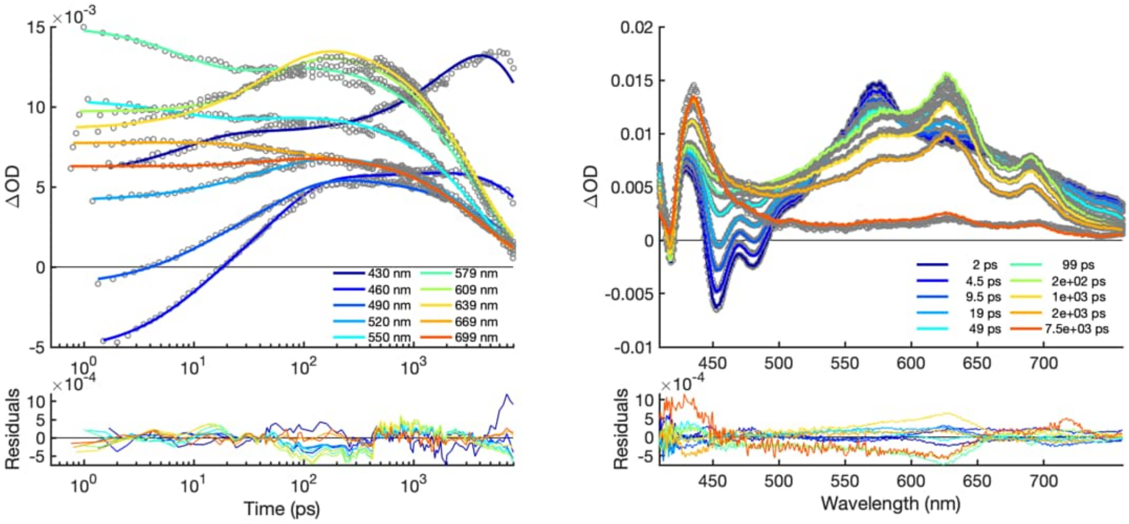

Figure S17. UV-vis TA spectroscopy of **2** in toluene at 220 K at selected wavelengths (left) and selected times (right). Time constants obtained from global analysis are τ_1 = 8 ps, τ_2 = 53 ps, τ_3 = 3.79 ns, and τ_4 >> 8 ns.

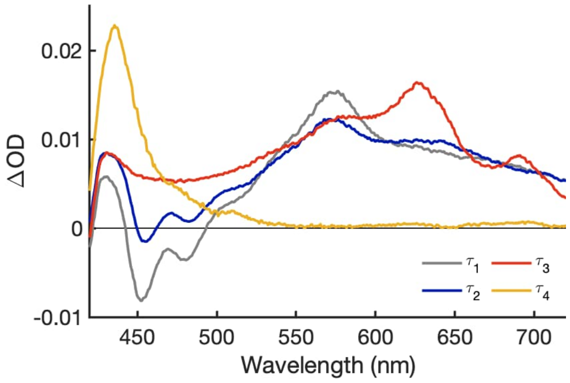

Figure S18. EAS obtained from global analysis for **2** in toluene at 220 K.

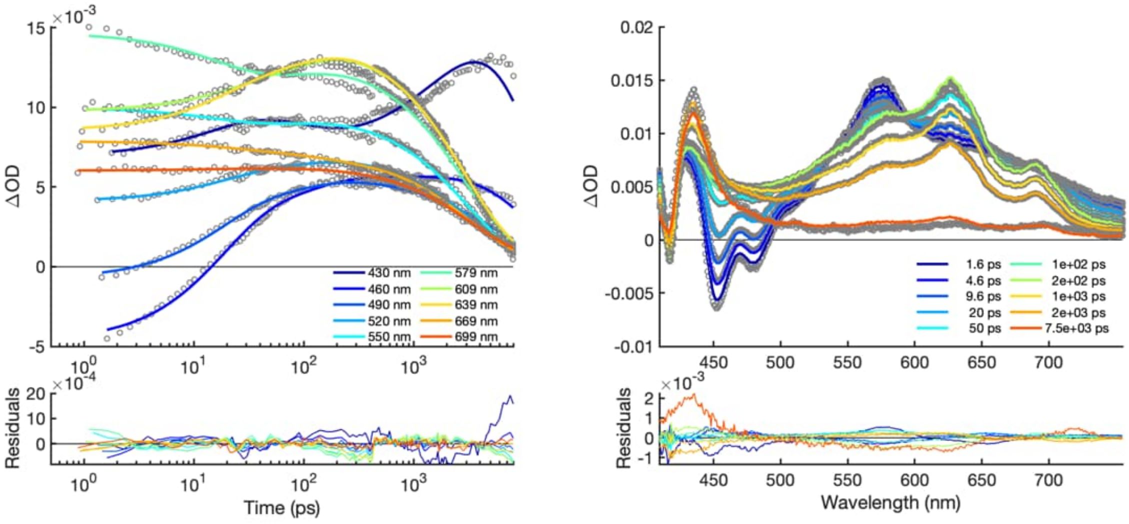

Figure S19. UV-vis TA spectroscopy of **2** in toluene at 240 K at selected wavelengths (left) and selected times (right). Time constants obtained from global analysis are τ_1 = 17 ps, τ_2 = 94 ps, τ_3 = 2.78 ns, and τ_4 >> 8 ns.

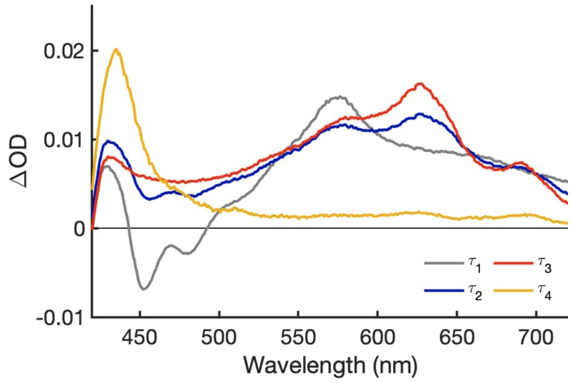

Figure S20. EAS obtained from global analysis for **2** in toluene at 240 K.

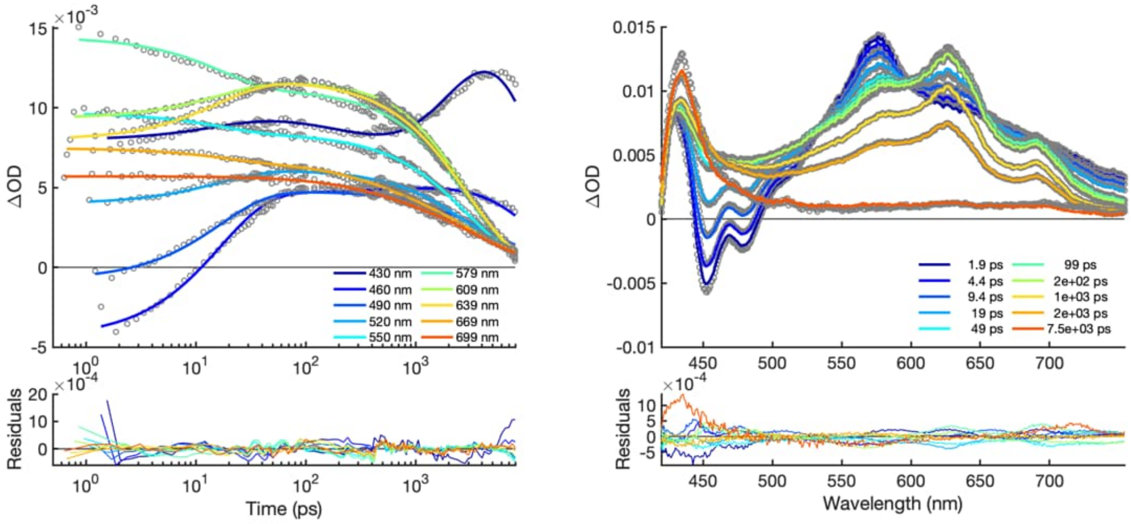

Figure S21. UV-vis TA spectroscopy of **2** in toluene at 260 K at selected wavelengths (left) and selected times (right). Time constants obtained from global analysis are τ_1 = 17 ps, τ_2 = 409 ps, τ_3 = 2.83 ns, and τ_4 >> 8 ns.

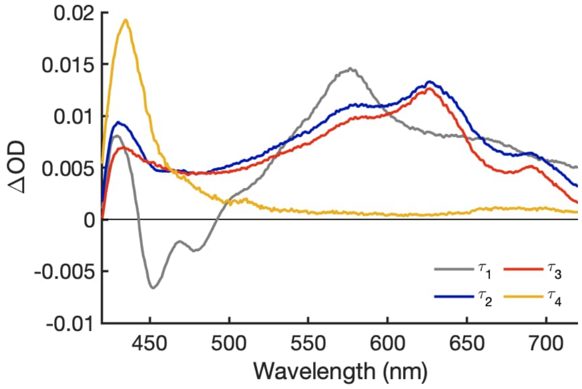

Figure S22. EAS obtained from global analysis for **2** in toluene at 260 K.

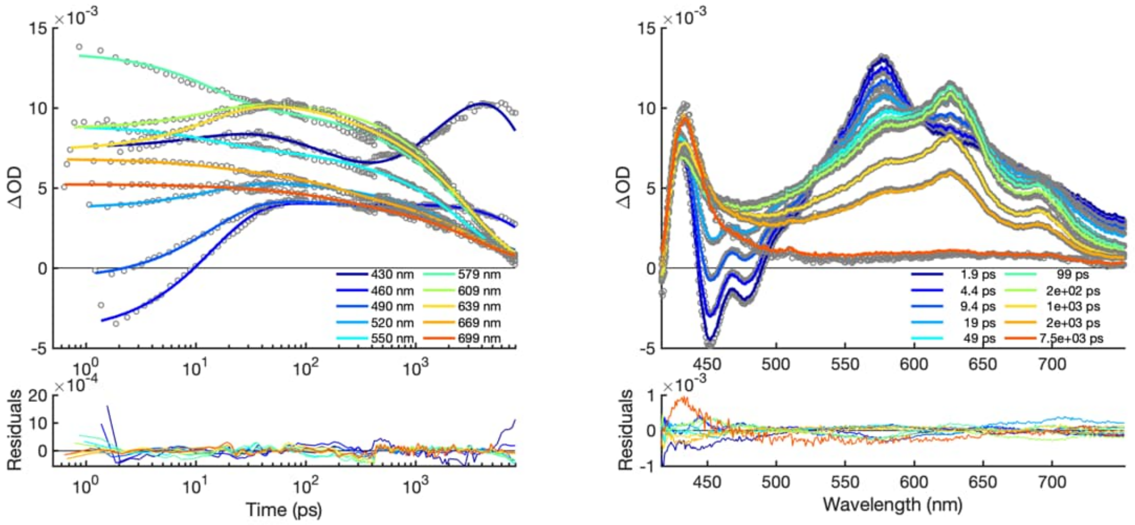

Figure S23. UV-vis TA spectroscopy of **2** in toluene at 280 K at selected wavelengths (left) and selected times (right). Time constants obtained from global analysis are τ_1 = 14 ps, τ_2 = 191 ps, τ_3 = 2.80 ns, and τ_4 >> 8 ns.

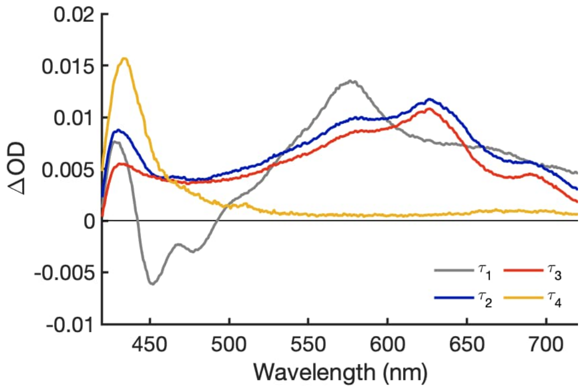

Figure S24. EAS obtained from global analysis for **2** in toluene at 280 K.

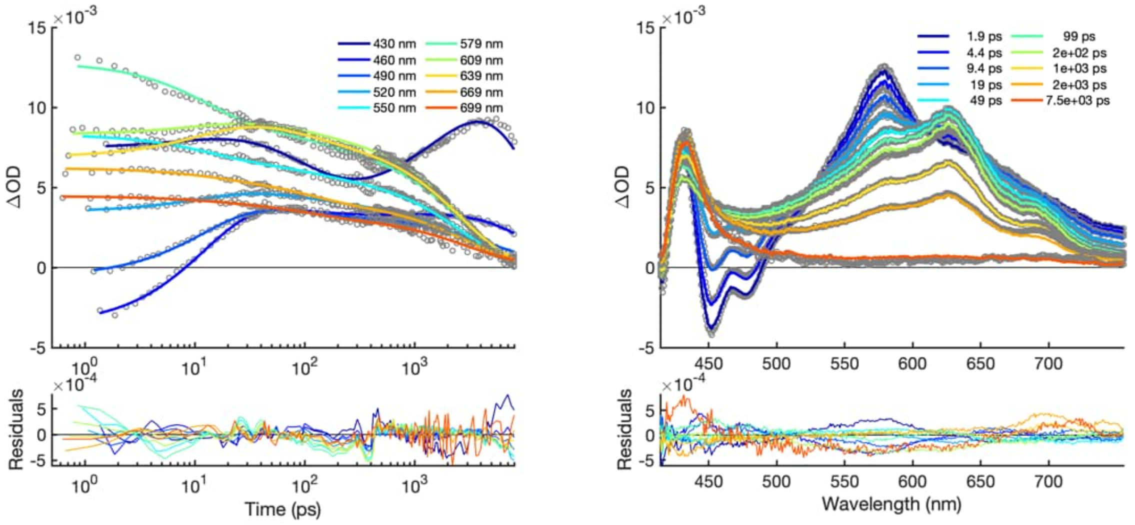

Figure S25. UV-vis TA spectroscopy of **2** in toluene at 298 K at selected wavelengths (left) and selected times (right). Time constants obtained from global analysis are τ_1 = 12 ps, τ_2 = 103 ps, τ_3 = 2.54 ns, and τ_4 >> 8 ns. Note that this data was also reported in reference [1].

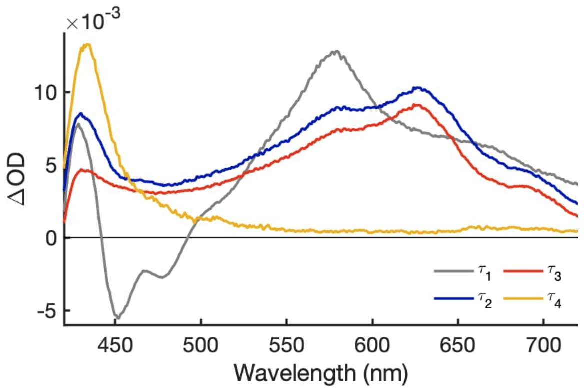

Figure S26. EAS obtained from global analysis for **2** in toluene at 298 K.

1.3 Triad **3** Time Traces, Spectra, and EAS

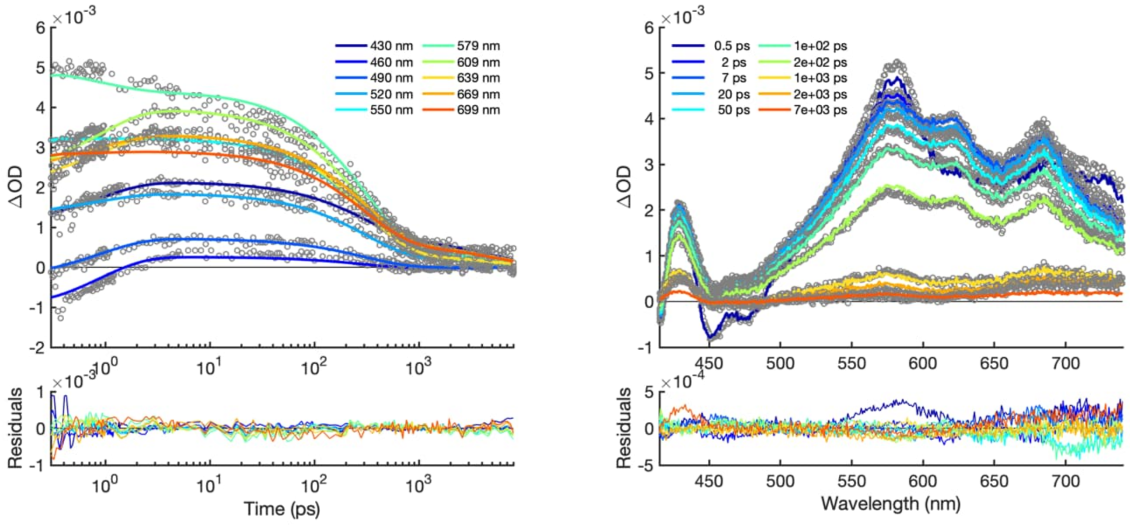

Figure S27. UV-vis TA spectroscopy of **3** in toluene at room temperature at selected wavelengths (left) and selected times (right). Time constants obtained from global analysis are τ_1 = 0.9 ps, τ_2 = 301 ps, and τ_3 = 5.9 ns.

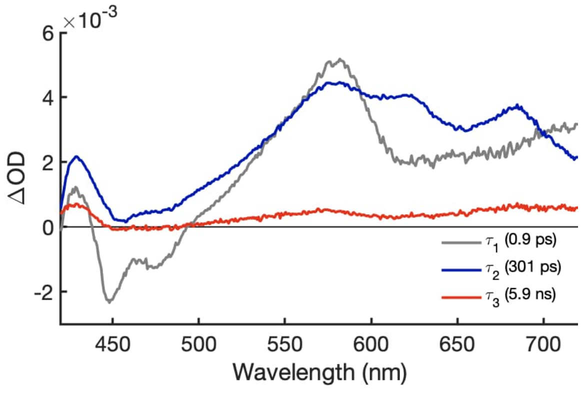

Figure S28. EAS obtained from global analysis for **3** in toluene at room temperature. The second component (blue trace) shows the characteristic spectral features of the CSS (peak at ~425 nm assigned to a phenoxyl radical, and broad absorbance with peaks at ~625 nm and ~675 nm, assigned to an anthracene radical anion).

The low solubility of triad **3** at lower temperatures precluded further measurements.

2 Formation Yields, Branching Ratios, and Target Analysis

Concentrations of ^{1*}An, CSS, and ^{3*}An were estimated using absorption values from TA spectra amplitudes and extinction coefficients for the three species. The extinction coefficients used for ^{1*}An ($\varepsilon_{580\ nm}$ ~7500 cm^{-1}mol^{-1}L)[2] and ^{3*}An ($\varepsilon_{435\ nm}$ ~75000 cm^{-1}mol^{-1}L)[3] were obtained from the literature. The value for the CSS ($\varepsilon_{625\ nm}$ ~7500 cm^{-1}mol^{-1}L), was estimated from prior UV-vis spectro-electrochemical measurements to generate the anthracene radical anion[4] and the literature ε value of for ground state anthracene (ε_{410nm} ~ 10000 cm^{-1}mol^{-1}L).[5,6] From the concentrations, yields were calculated according to eq 1 and eq 2 (see main text).

Uncertainty in the formation yields was considered from multiple sources. The extinction coefficient for ^{1*}An was reported with an uncertainty of ± 2500 cm^{-1}mol^{-1}L.[2] Assuming ^{1*}An extinction coefficient to be 10,000 cm^{-1}mol^{-1}L instead of 7500 cm^{-1}mol^{-1}L, the yields increase by 33%. Therefore, we have assigned an absolute error of ± 33% for the formation yields. However, since the same extinction coefficients are used for all calculations, the relative yield is much lower and estimated at ± 10%. This value comes from assuming the TA amplitudes have an uncertainty of ± 5%, which results in a ± 10% variation in formation yield.

Target analysis was performed as previously described to model two branching pathways from the ^{1}CSS: inverted CR to the ground state (GS) or formation of ^{3*}An (Figure S29).[1] The rate constant assignments are as follows: k_1 corresponds to vibrational relaxation of the ^{1*}An$_{(hot)}$, k_2 corresponds to CS from the relaxed ^{1*}An$_{(relaxed)}$ to the CSS, k_3 represents to the overall rate of decay of the CSS, and k_4 represents the decay of ^{3*}An. The constants b_1 and b_2 are the branching ratios (weight parameters) for decay of the CSS to the GS and decay of the CSS to form ^{3*}An, respectively. The extracted rate for the individual pathways can be obtained by multiplying the overall rate by the pathway's branching ratio. The rate of CS is therefore equal to k_3*b_1 and the rate of ^{3*}An formation is k_3*b_2. The branching ratios are calculated from concentrations of the CSS and ^{3*}An such that b_1 = ([CSS] – [^{3*}An])/[CSS] and b_2 = 1–b_1 and are given in Table S1.

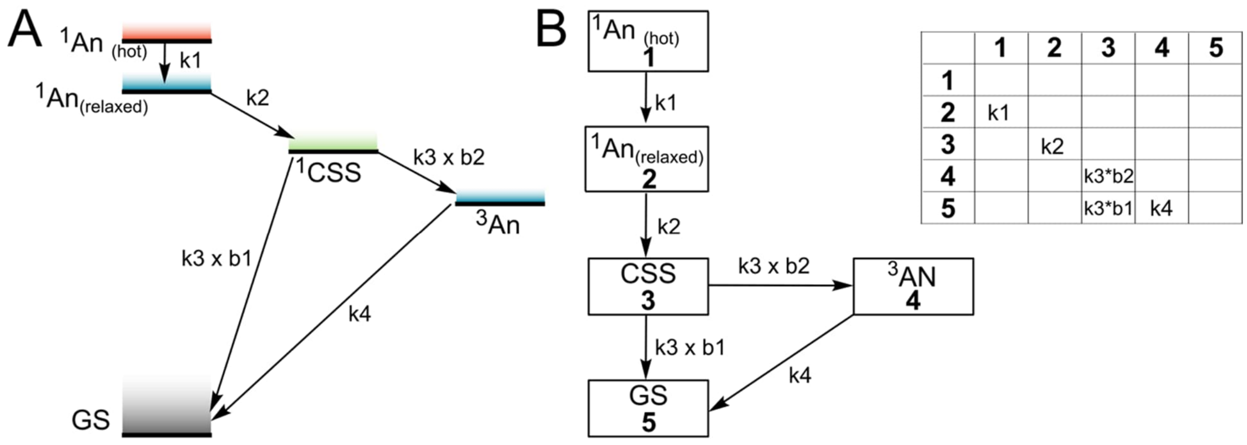

Figure S29. Reaction model used in target analysis. A) Jablonski diagram of the states that are observable by TA spectroscopy and relevant transitions between them. B) Compartment model and K-matrix used in target analysis. Reproduced with permission from reference 1. Copyright 2021 American Chemical Society.

Table S1. Branching ratios for reaction pathways from the CSS. b_1 corresponds to the GS pathway and b_2 corresponds to ^{3*}An formation pathway.

	**1**		**2**	
T **(K)**	b_1	b_2	b_1	b_2
298	0.98	0.02	0.93	0.08
280	0.98	0.02	0.91	0.09
260	0.97	0.03	0.87	0.13
240	0.92	0.08	0.86	0.14
220	0.91	0.09	0.86	0.14
200	0.89	0.11	0.87	0.13
185	0.89	0.11	0.85	0.15

3 Arrhenius Analysis with Extracted Individual Rate Constants

Rate constants for CR (k_{CR}) were calculated as $k_{CR} = k_{CSS} \cdot b_1$, where k_{CSS} is from global analysis (Table 1 in the main text) and b_1 is the pathway branching ratio (S1). To calculate rate constants for reactions from ^{1*}An, two different pathways from ^{1*}An were considered: PCEnT to form *[PhO=pyH] and CS to form the CSS. Branching ratios for the two pathways were calculated from concentrations of ^{1*}An and CSS, where b_3 corresponds to the PCEnT pathway and b_4 corresponds to CS (Table S2). The relationships used were $b_3 = ([^{1*}\text{An}] - [\text{CSS}])/[^{1*}\text{An}]$ and $b_4 = 1 - b_3$. The rate constant of PCEnT (k_{PCEnT}) was calculated from $k_{PCEnT} = b_3 \cdot k_{LES}$ and the rate constant

for CS (k_{CS}) was calculated as $k_{CS} = b_4 \cdot k_{LES}$, where k_{LES} is from global analysis (Table 1 in the main text). Calculated rate constants are given in Table 3 in the main text and Arrhenius analysis was applied to CS, PCEnT, and CR (Figure S30 and Table S3). The individual rate constants for CSS deactivation in triad **1**, k_{CR} and k_{ET} reported in Table 3 of the main text, were calculated from the Arrhenius parameters of Table S3, subtracting the contribution from CR to generate the ^{3*}An. The latter rate constant is $k_T = 3 \cdot 10^7$ s^{-1} in **2,** at all temperatures, as given by $k_T = \left(\Phi_{^{3*}\mathrm{An}} / \Phi_{CSS} \right) / \tau_3$. For **1**, the value of k_T at 185 K is the same as for **2**, but varies with temperature, probably because of the greater uncertainty in quantum yields at higher temperature as the triplet yields become very small.

Table S2. Branching ratios for reaction pathways from ^{1*}An. b_3 corresponds to the PCEnT pathway and b_4 corresponds to CS pathway.

	**1**		**2**	
***T* (K)**	b_3	b_4	b_3	b_4
298	0.74	0.26	0.10	0.90
280	0.71	0.29	0.08	0.92
260	0.61	0.39	0.20	0.80
240	0.68	0.32	0.11	0.89
220	0.67	0.33	0.12	0.88
200	0.67	0.33	0.16	0.84
185	0.66	0.34	0.19	0.81

Table S3. Activation energies and preexponential factors from Arrhenius analyses

**Reaction**	**Triad**	E_a **(meV)**	A **(s^{-1})**
**CS**	**1**	61 ± 8	1.0 (± 0.4) × 10^{11}
	**2**	110 ± 10	6 (± 3) × 10^{12}
**PCEnT**	**1**	70 ± 1	3.5 (± 0.2) × 10^{11}
	**2**	80 ± 19	2 (± 2) × 10^{11}
**CR**	**1** (T ≥ 240)	240 ± 10	8 (± 4) × 10^{13}
	**1** (T ≤ 220)	37 ± 17	2 (± 2) × 10^{9}
	**2**	26 ± 3	1.0 (± 2) × 10^{9}

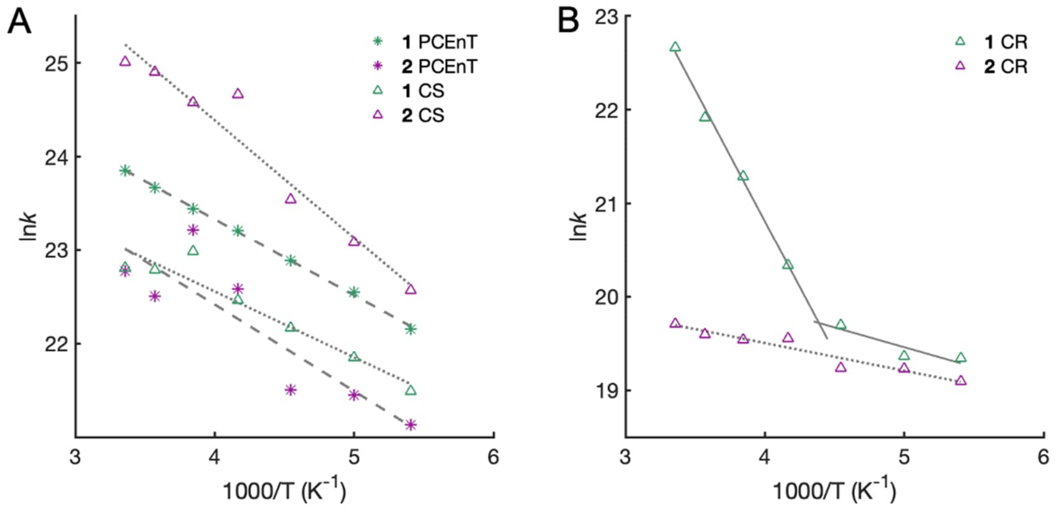

Figure S30. Arrhenius analysis for **1** (green) and **2** (purple) using extracted rates. A) Extracted rates for k_{PCEnT} (stars) and k_{CS} (triangles). B) Extracted rates for k_{CR} (triangles).

References

1. Cotter, L. F.; Rimgard, B. P.; Parada, G. A.; Mayer, J. M.; Hammarström, L. Solvent and Temperature Effects on Photoinduced Proton-Coupled Electron Transfer in the Marcus Inverted Region. *J. Phys. Chem. A* **2021,** *125*, 7670-7684.
2. Bachilo, S. M. Absorption from excited singlet states of anthracene and 1,2-benzanthracene. *J. Appl. Spectrosc.* **1993,** *58*, 99-103.
3. Dempster, D. N.; Morrow, T.; Quinn, M. F. Extinction coefficients for triplet-triplet absorption in ethanol solutions of anthracene, naphthalene, 2,5-diphenyloxazole, 7-diethylamino- 4-methyl coumarin and 4-methyl-7-amino-carbostyril. *J. Photochem.* **1973,** *2*, 329-341.
4. Parada, G. A.; Goldsmith, Z. K.; Kolmar, S.; Pettersson Rimgard, B.; Mercado, B. Q.; Hammarström, L.; Hammes-Schiffer, S.; Mayer, J. M. Concerted proton-electron transfer reactions in the Marcus inverted region. *Science* **2019,** *364*, 471.
5. Glöcklhofer, F.; Rosspeintner, A.; Pasitsuparoad, P.; Eder, S.; Fröhlich, J.; Angulo, G.; Vauthey, E.; Plasser, F. Effect of symmetric and asymmetric substitution on the optoelectronic properties of 9,10-dicyanoanthracene. *Mol. Syst. Des. Eng.* **2019,** *4*, 951-961.
6. Jones, R. N. The Ultraviolet Absorption Spectra of Anthracene Derivatives. *Chem. Rev.* **1947,** *41*, 353-371.
